# *Fusobacterium nucleatum* Promotes Colorectal Cancer Cell to Acquire Stem Cell‐Like Features by Manipulating Lipid Droplet‐Mediated Numb Degradation

**DOI:** 10.1002/advs.202105222

**Published:** 2022-02-15

**Authors:** Haiyang Liu, Junfeng Du, Shanshan Chao, Shuoguo Li, Huiyun Cai, Hongjie Zhang, Gang Chen, Pingsheng Liu, Pengcheng Bu

**Affiliations:** ^1^ Key Laboratory of RNA Biology Key Laboratory of Protein and Peptide Pharmaceutical Institute of Biophysics Chinese Academy of Sciences Beijing 100101 China; ^2^ Department of General Surgery the 7th Medical Center Chinese PLA General Hospital Beijing 100700 China; ^3^ The 2nd School of Clinical Medicine Southern Medical University Guangdong 510515 China; ^4^ Medical Department of General Surgery the 1st Medical Center Chinese PLA General Hospital Beijing 100853 China; ^5^ College of Life Sciences University of Chinese Academy of Sciences Beijing 100049 China; ^6^ Center for Biological Imaging Institute of Biophysics Chinese Academy of Sciences Beijing 100101 China; ^7^ The core facility Institute of Biophysics Chinese Academy of Sciences Beijing 100101 China; ^8^ National Laboratory of Biomacromolecules Institute of Biophysics Chinese Academy of Sciences Beijing 100101 China; ^9^ Center for Excellence in Biomacromolecules Chinese Academy of Sciences Beijing 100101 China

**Keywords:** colorectal cancer, colorectal cancer stem‐like cells, *Fusobacterium nucleatum*, lipid droplets, numb

## Abstract

*Fusobacterium nucleatum* is a critical microbe that contributes to colorectal cancer progression and chemoresistance. However, whether and how *F. nucleatum* regulates colorectal cancer stem‐like cells (CCSCs) remains unknown. Here, the authors show that *F. nucleatum* promotes CCSC self‐renewal, and non‐CCSCs to acquire CCSC features by manipulating cellular lipid accumulation. *F. nucleatum* infection decreases lipid accumulation in CCSCs by enhancing fatty acid oxidation, thus promoting CCSC self‐renewal. In contrast, *F. nucleatum* increases lipid accumulation in non‐CCSCs by promoting fatty acid formation. Lipids are deposited as lipid droplets, which recruits Numb, a key cell fate regulator, through the AP2A/ACSL3 complex, and MDM2, an E3 ubiquitin ligase, though VCP and UBXD8. On lipid droplets, Numb is degraded by MDM2, activating Notch signaling, thus promoting gain of stem‐like cell features. Their findings demonstrate that *F. nucleatum* directly manipulates colorectal cancer cell fate and reveal the mechanism of lipid droplet‐mediated Numb degradation for activating Notch signaling.

## Graphical abstract

*Fusobacterium nucleatum* increases lipid accumulation in non‐CCSCs by promoting fatty acid formation. Lipids are deposited as lipid droplets, which recruiting Numb through the AP2A/ACSL3 complex, and MDM2, an E3 ubiquitin ligase, though VCP and UBXD8. On lipid droplets, Numb is degraded by MDM2, activating Notch signaling, thus promoting gain of stem‐like cell features.

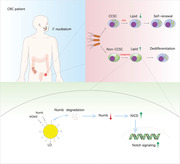

## 1 Introduction

Despite remarkable advances in diagnosis and therapeutics, cancer remains among the leading causes of death globally. The cause is linked to a subpopulation of cells termed cancer stem‐like cells (CSCs), which likely originate from normal stem cells and are responsible for cancer initiation, progression, chemoresistance, and recurrence.^[^ ^1^ ^]^ Furthermore, emerging evidence shows that non‐CSCs are able to acquire stem‐like features during certain processes such as epithelial‐mesenchymal transition, expressing stem cell markers, efficiently forming spheres in vitro, and developing tumors in vivo.^[^ ^1^, ^2^ ^]^ Ranked as one of the most common malignancies and leading causes of cancer‐related deaths, colorectal cancer (CRC) has been experimentally shown to harbor colorectal cancer stem‐like cells (CCSCs), characterized by expressing stem cell markers such as ALDH1, CD133, and CD44.^[^ ^3^ ^]^ However, the mechanism of sustaining CCSC characteristics and reprogramming non‐CCSCs remains unclear, thus, largely accounting for the failure to treat CRC.

Lipid metabolism exerts a critical impact on maintaining CSC properties by balancing the activity between lipid synthesis and degradation.^[^ ^4^ ^]^ Catalyzed by fatty acid synthase (FASN), de novo fatty acids bind to a glycerol backbone to generate triglycerides, whose accumulation is stored as lipid droplets to protect against lipotoxicity and serve as energy storage.^[^ ^5^ ^]^ Conversely, fatty acid oxidation (FAO) decreases lipid accumulation and fuels CSC self‐renewal and proliferation using ATP.^[^ ^4^, ^6^ ^]^ In addition to storing triglycerides, lipid droplet‐associated proteins have been observed to degrade, mediated by a ubiquitin‐proteasome system, to manipulate lipid droplet homeostasis.^[^ ^7^ ^]^ However, it remains unknown whether non‐lipid droplet proteins can be degraded by the lipid droplet‐mediated ubiquitin‐proteasome system, and in turn, regulate cell fate‐associated signaling pathways.

Numerous studies have shown the important effects of microbes in CRC development. *Fusobacterium nucleatum* is one of the most well‐studied microbes, playing an important role in CRC tumorigenesis and progression. *F. nucleatum* levels are elevated in CRC tissues compared to normal controls and the enriched abundance is associated with poor prognosis and shortened survival.^[^ ^8^ ^]^ Additional studies have demonstrated that *F. nucleatum* promotes colorectal carcinogenesis and progression by manipulating the immune microenvironment, recruiting tumor‐infiltrating lymphocytes, protecting CRC cells from immune cell attack, inducing resistance to chemotherapy, and activating signaling pathways such as NF‐*κ*B.^[^ ^8^, ^9^ ^]^ However, the potential regulation of *F. nucleatum* on CCSCs has not been investigated. In this study, we find that *F. nucleatum* infection promotes CCSC self‐renewal, and non‐CCSCs to acquire stem‐like cell features by manipulating lipid accumulation. Specifically, we demonstrate a novel role for lipid droplets in non‐CCSCs. We show that lipid droplets recruit Notch inhibitor Numb and E3 ubiquitin‐protein ligase MDM2 to their surface, facilitating MDM2‐mediated Numb degradation and in turn activating Notch signaling, which promotes non‐CCSCs to acquire stem‐like features.

## 2 Results

### 2.1 *F. nucleatum* Enhances CCSC Self‐Renewal and Xenograft Tumorigenicity

**Figure 1:**
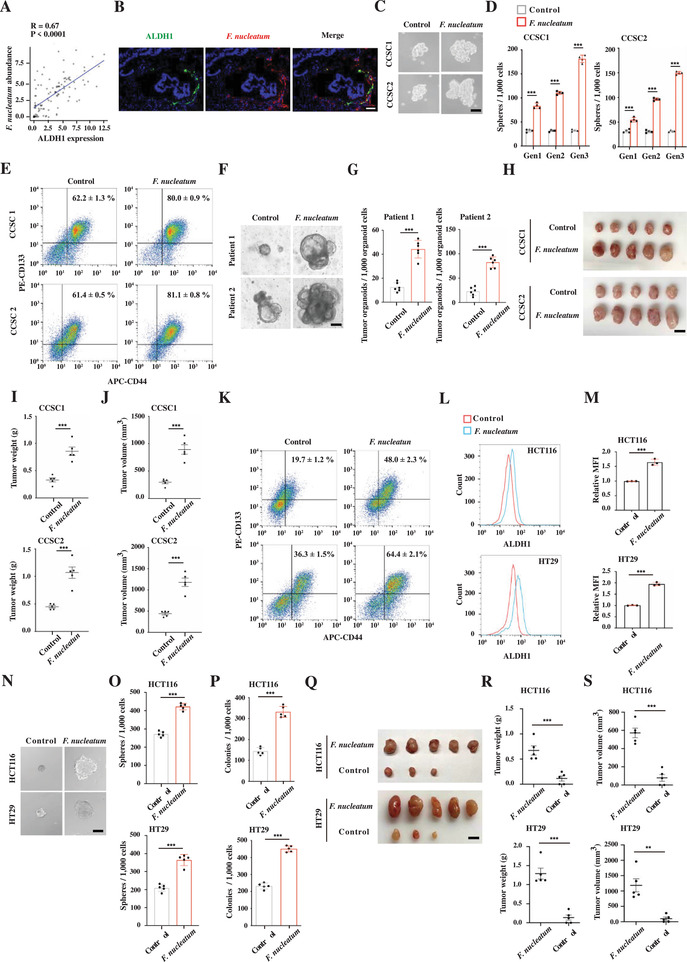
*F. nucleatum* enhances CCSC self‐renewal and promotes gain of stem cell‐like features in non‐CCSCs. A) Real‐time PCR showing the correlation of *F. nucleatum* abundance and ALDH1 expression in CRC tissues. *p*‐Value was calculated using Pearson correlation analysis (*n* = 103). B) Representative images of fluorescence in situ hybridization showing that *F. nucleatum* infected both ALDH1 positive and negative cells in CRC tissues. Scale bar, 50 µm. C,D) Representative images (C) and quantification (D) of CCSC sphere formation during serial passages after *F. nucleatum* infection. The representative images are spheres at the second generation. Error bars denote the s.d. (*n* = 4 per group). Gen, generation. *p*‐Value was calculated using one‐way ANOVA with post‐hoc test. ***, *p* < 0.001. Scale bar, 100 µm. E) Flow cytometry showing CD133^+^CD44^+^ CCSCs in the control and *F. nucleatum*‐infected CCSC sphere cells. F,G) Representative images (F) and quantification (G) of CRC organoids cultured with or without *F. nucleatum*. One thousand organoid cells from each condition were reseeded to examine organoid formation and growth. Error bars denote the s.d. (*n* = 6 per group). *p*‐Value was calculated using Student's *t*‐test. ***, *p* < 0.001. Scale bar, 100 µm. H–J) Images (H), weight (I), and volume (J) of xenograft tumors developed from the control and *F. nucleatum* infected CCSC sphere cells. Error bars denote the s.e.m. (*n* = 5 per group). *p*‐Value was calculated using Student's *t*‐test. ***, *p* < 0.001. Scale bar, 1 cm. K) Flow cytometry showing the percentages of CD133^+^CD44^+^ CCSCs in xenograft tumors in (H–J). L,M) Representative histogram (L) and quantification (M) of flow cytometry showing ALDH1 expression in the control and *F. nucleatum*‐infected HCT116 and HT29 cells. The red and blue histograms represent uninfected and infected cells, respectively. Error bars denote the s.d. (*n* = 3 per group). *p*‐Value was calculated using Student's *t*‐test. ***, *p* < 0.001. N,O) Representative images (N) and quantification (O) of sphere formation in the control and *F. nucleatum*‐infected HCT116 and HT29 cells. Error bars denote the s.d. (*n* = 5 per group). *p*‐Value was calculated using Student's *t*‐test. ***, *p* < 0.001. Scale bar, 100 µm. P) Clonogenicity assay of the control and *F. nucleatum*‐infected HCT116 and HT29 cells. Error bars denote the s.d. (*n* = 5 per group). *p*‐Value was calculated using Student's *t*‐test. ***, *p* < 0.001. Q–S) Images (Q), weight (R), and volume (S) of xenograft tumors developed from the control and *F. nucleatum*‐infected HCT116 and HT29 cells. Error bars denote s.e.m. (*n* = 5 per group). *p*‐Value was calculated using Student's *t*‐test. **, *p* < 0.01; ***, *p* < 0.001. Scale bar, 1 cm.

To investigate the potential regulation between *F. nucleatum* and CCSCs, we analyzed the abundance of both populations in frozen CRC tissues collected from 103 patients by measuring levels of the *F. nucleatum* 16S rRNA gene and the expression of CCSC marker gene ALDH1. We found that *F. nucleatum* abundance was positively correlated with the expression of ALDH1 (**Figure** **1**A). We further prepared an *F. nucleatum* antibody to visualize how *F. nucleatum* infected the cells in CRC tissue by immunostaining (Figure S1A, Supporting Information), finding that *F. nucleatum* infected both ALDH1 positive and negative cells (Figure 1B). Furthermore, the accumulation of *F. nucleatum* around ALDH1 positive cells is likely due to hypoxic conditions (Figure S1B, Supporting Information). Thus, it is possible that *F. nucleatum* directly influences CCSC behavior.

Previously, we isolated CCSCs from two CRC patients using CCSC markers ALDH1, CD133, and CD44, and characterized the stem‐like features by the sphere propagation assay and limiting dilution assay.^[^ ^10^ ^]^ We then co‐cultured the CCSC sphere cells with *F. nucleatum* to determine whether *F. nucleatum* regulates CCSC self‐renewal. We observed that *F. nucleatum* significantly enhanced the CCSC sphere‐formatting capability, showing larger and more numerous spheres during serial propagation (Figure 1C,D and Figure S1C, Supporting Information). In addition, *F. nucleatum* infection upregulated the expression of many other CCSC marker genes as well as the CD133^+^CD44^+^ CCSC proportion in the spheres (Figure 1E and Figure S1D, Supporting Information). Cancer organoids are able to recapitulate the original cancer characteristics and the efficiency of the cancer organoid‐forming represents CSC self‐renewal capability.^[^ ^11^ ^]^ To test whether *F. nucleatum* promotes CSC self‐renewal in cancer organoids, we co‐cultured it with CRC cells freshly dissected from CRC tissues. We found that *F. nucleatum* does facilitate CRC organoid formation; in fact, *F. nucleatum*‐infected cells developed much more, larger organoids when reseeded equally with control organoid cells, suggesting that *F. nucleatum* infection expanded CCSCs in the CRC organoids (Figure 1F,G). To determine the influence of *F. nucleatum* on CCSC tumorigenicity, we infected CCSCs with *F. nucleatum* and transplanted the cells subcutaneously into nude mice. We observed that *F. nucleatum* promoted CCSC xenograft formation and elevated the CD133^+^CD44^+^ CCSC proportion in the xenograft tumors (Figure 1H–K). Together, these data suggest that *F. nucleatum* promotes CCSC self‐renewal and tumorigenicity.

### 2.2 *F. nucleatum* Promotes Non‐CCSCs to Acquire Stem‐Like Features

Notably, immunostaining showed that *F. nucleatum* infected non‐CCSCs as well as CCSCs (Figure 1B), suggesting a role in non‐CCSC regulation. To investigate how *F. nucleatum* influences non‐CCSCs, we removed the pre‐existing ALDH1+ cells from CRC cells HCT116 and HT29 (Figure S1E, Supporting Information), infected the cells with *F. nucleatum* and examined stem cell marker expression, sphere propagation, clonogenicity, and xenograft tumor development. We found that *F. nucleatum* significantly upregulated the expression of stem cell makers Lgr5, Olfm4, Sox9, and Aldh1. Conversely, *F. nucleatum* downregulated the expression of differentiation marker Cdx2 (Figure 1L,M and Figure S1F, Supporting Information). Furthermore, *F. nucleatum* significantly increased the capability of sphere propagation, clonogenicity, and xenograft tumor growth (Figure 1N–S). Together, the data suggest that non‐CCSCs possess stem‐like cell properties when infected with *F. nucleatum*.

### 2.3 *F. nucleatum* Decreases Lipid Accumulation in CCSCs by Enhancing Fatty Acid Oxidation

**Figure 2:**
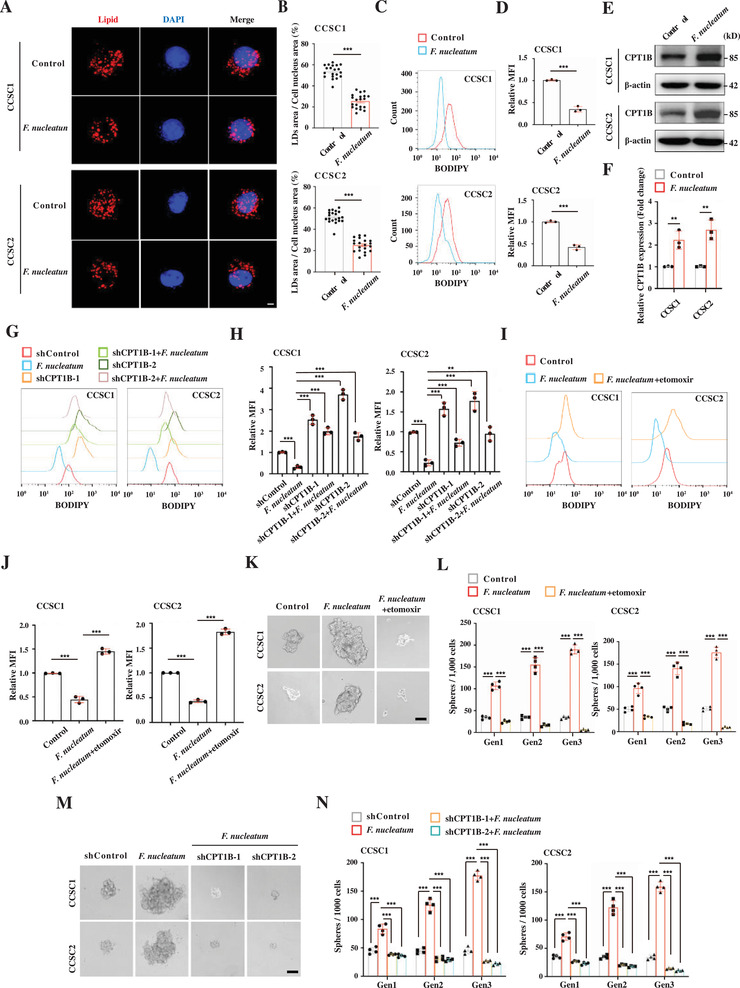
*F. nucleatum* promotes CCSC self‐renewal by enhancing CPT1B‐mediated fatty acid oxidation. A,B) Representative 3D‐SIM images (A) and quantification (B) of lipid droplets (LDs) contained in the control and *F. nucleatum*‐infected CCSCs. Lipids were stained with Nile red. Cells from each group were randomly selected and quantified (*n* = 20 per group). Scale bar, 5 µm. Error bars denote s.e.m. *p*‐Value was calculated using Student's *t*‐test. ***, *p* < 0.001. C,D) Representative histogram (C) and quantification (D) of flow cytometry with BODIPY staining showing lipid content in the control and *F. nucleatum*‐infected CCSC sphere cells. The red and blue histograms represent uninfected controls and infected cells, respectfully. Error bars denote the s.d. (*n* = 3 per group). *p*‐Value was calculated using Student's *t*‐test. ***, *p* < 0.001. E,F) Representative Western blot imaging (E) and quantification (F) showing CPT1B expression in the control and *F. nucleatum*‐infected CCSC sphere cells. Error bars denote the s.d. (*n* = 3 per group). *p*‐Value was calculated using one‐way ANOVA with post‐hoc test. **, *p* < 0.01. G,H) Representative histogram (G) and quantification (H) of flow cytometry with BODIPY staining showing lipid levels in the control, CPT1B knockdown (shCPT1B‐1 and shCPT1B‐2), *F. nucleatum*‐infected, and *F. nucleatum*‐infected with CPT1B knockdown (shCPT1B‐1 and shCPT1B‐2) CCSC sphere cells. Error bars denote the s.d. (*n* = 3 per group). *p*‐Value was calculated using one‐way ANOVA with post‐hoc test. **, *p* < 0.01; ***, *p* < 0.001. I,J) Representative histogram (I) and quantification (J) of flow cytometry with BODIPY staining showing lipid levels in the control, *F. nucleatum*‐infected, and *F. nucleatum*‐infected with etomoxir treatment (50 µm) CCSC sphere cells. Error bars denote the s.d. (*n* = 3 per group). *p*‐Value was calculated using one‐way ANOVA with post‐hoc test. ***, *p* < 0.001. K,L) Representative images (K) and quantification (L) of sphere formation of the control, *F. nucleatum*‐infected, and *F. nucleatum*‐infected with etomoxir treatment (50 µm) CCSC sphere cells. Error bars denote the s.d. (*n* = 4 per group). *p*‐Value was calculated using one‐way ANOVA with post‐hoc test. ***, *p* < 0.001. Scale bar, 100 µm. M,N) Representative images (M) and quantification (N) of sphere formation of the control, *F. nucleatum*‐infected, and *F. nucleatum*‐infected with CPT1B knockdown (shCPT1B‐1 and shCPT1B‐2) CCSC sphere cells. Error bars denote the s.d. (*n* = 4 per group). *p*‐Value was calculated using one‐way ANOVA with post‐hoc test. ***, *p* < 0.001. Scale bar, 100 µm.

Increasing evidence has shown that alterations in lipid metabolism, including lipid accumulation, closely contribute to stemness maintenance.^[^ ^12^ ^]^ To determine whether *F. nucleatum* regulates lipid accumulation in CCSCs, we infected CCSCs with *F. nucleatum* and evaluated lipid levels by staining with Nile red. We found that neutral lipids were highly accumulated in CCSCs, while their levels were decreased upon *F. nucleatum* infection (**Figure** **2**A,B). Flow cytometry with BODIPY staining further confirmed that *F. nucleatum* downregulated lipid droplets in CCSCs (Figure 2C,D).

To understand the mechanism by which *F. nucleatum* regulates neutral lipid accumulation in CCSCs, we analyzed the transcriptome of *F. nucleatum*‐infected CCSCs and the uninfected controls by performing RNA‐seq. Gene ontology showed that *F. nucleatum* highly altered pathways involved in the lipid catabolic process (Figure S2A, Supporting Information). Among the *F. nucleatum*‐regulated lipid catabolic genes, carnitine palmitoyltransferase 1B (CPT1B) was significantly upregulated in CCSCs, which was further validated by RT‐qPCR and Western blot (Figure 2E,F and Figures S2B and S2C, Supporting Information). CPT1B is the key metabolic enzyme that catalyzes the rate‐limiting step of FAO, resulting in a reduction of neutral lipid accumulation.^[^ ^13^ ^]^ Therefore, we hypothesized that CPT1B‐mediated FAO likely contributed to the reduction of lipid accumulation in CCSCs. Consistent with this hypothesis, we found that CPT1B knockdown significantly abrogated *F. nucleatum*‐reduced neutral lipid accumulation (Figure 2G,H and Figure S3A, Supporting Information). Similar results were observed when FAO was specifically inhibited by etomoxir (Figure 2I,J). Together, the data suggest that *F. nucleatum* decreases lipid droplets in CCSCs, likely by enhancing CPT1B‐mediated FAO.

### 2.4 CPT1B‐Mediated Fatty Acid Oxidation Enhances CCSC Self‐Renewal

*F. nucleatum* infection upregulated the expression of CPT1B, the rate‐limiting enzyme of FAO in CCSCs, and increasing evidence has shown that FAO is important for maintaining stemness and proliferation of CSCs, such as breast cancer stem‐like cells and quiescent leukemia progenitor cells.^[^ ^4^, ^14^ ^]^ Thus, we hypothesized that *F. nucleatum* infection might enhance FAO rate in CCSCs, which in turn would promote proliferation and self‐renewal of CCSCs. To test this hypothesis, we treated CCSCs with ^3^H‐oleic acid and examined the FAO rate by measuring ^3^H_2_O production. We found that *F. nucleatum* infection indeed enhanced FAO in CCSCs, showing increased ^3^H_2_O levels (Figure S3B, Supporting Information). In addition, *F. nucleatum*‐infected CCSCs produced much higher levels of ATP compared with the control cells (Figure S3C, Supporting Information). Furthermore, we found that FAO inhibitor, etomoxir, significantly suppressed *F. nucleatum* infection‐enhanced CCSC self‐renewal (Figure 2K,L and Figure S3D, Supporting Information), and the suppressive effect was not related to the reactive oxygen‐species accumulation induced by etomoxir treatment (Figure S3E,F, Supporting Information). Consistently, we observed that CPT1B knockdown suppressed CCSC self‐renewal and proliferation as well (Figure 2M,N and Figure S3G, Supporting Information). It has been reported that lipid oxidation can result in ferroptosis, which might suppress cancer stem‐like cell self‐renewal.^[^ ^15^ ^]^ We then examined ferroptosis in *F. nucleatum*‐infected CCSCs and found that *F. nucleatum* infection did not cause ferroptosis in CCSCs (Figure S3H–J, Supporting Information). Taken together, these data show that *F. nucleatum* infection enhances the FAO rate in CCSCs, through which CCSCs gain an elevated self‐renewal capability.

### 2.5 *F. nucleatum* Increases Lipid Accumulation in Non‐CCSCs by Enhancing Lipid Fatty Acid Synthesis

**Figure 3:**
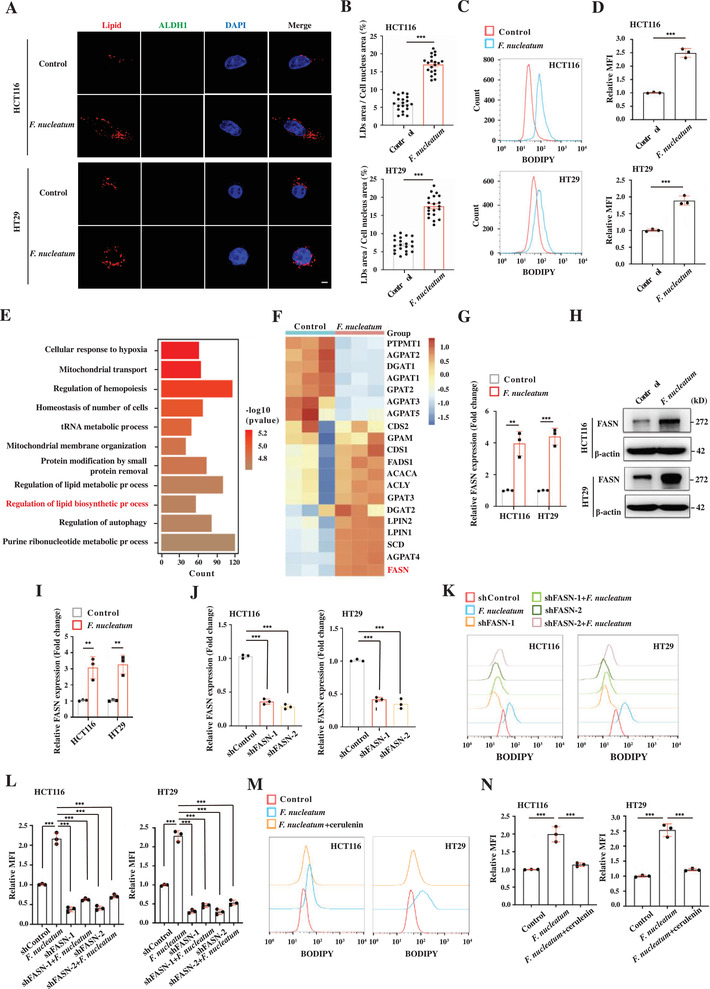
*F. nucleatum* increases lipid accumulation by enhancing FASN‐mediated fatty acid synthesis in CRC cells. A,B) Representative 3D‐SIM images (A) and quantification (B) of lipid droplets (LDs) contained in the control and *F. nucleatum*‐infected HCT116 and HT29 cells. Pre‐existing ALDH1+ cells have been removed before infection. Lipids were stained with Nile red. The cells were ALDH1 negative, shown by immunostaining with anti‐ALDH1 antibody. Cells from each group were randomly selected and quantified (*n* = 20 per group). Scale bar, 5 µm. Error bars denote s.e.m. *p*‐Value was calculated using Student's *t*‐test. ***, *p* < 0.001. C,D) Representative histogram (C) and quantification (D) of flow cytometry with BODIPY staining showing lipid content in the control and *F. nucleatum*‐infected HCT116 and HT29 cells. The red and blue histograms represent uninfected and infected cells, respectfully. Error bars denote the s.d. (*n* = 3 per group). *p*‐Value was calculated using Student's *t*‐test. ***, *p* < 0.001. E) Gene ontology analyses on the RNA‐seq dataset showing the differentially expressed genes in the control and *F. nucleatum*‐infected HCT116 cells. F) Heat map on the RNA‐seq dataset showing the differentially expressed genes in the control and *F. nucleatum* infected HCT116 cells. G) RT‐qPCR showing FASN expression in the control and *F. nucleatum*‐infected HCT116 and HT29 cells. Error bars denote s.d. (*n* = 3 per group). *p*‐Value was calculated using one‐way ANOVA with post‐hoc test. **, *p* < 0.01; ***, *p* < 0.001. H,I) Representative Western blot imaging (H) and quantification (I) showing FASN expression in the control and *F. nucleatum*‐infected HCT116 and HT29 cells. Error bars denote s.d. (*n* = 3 per group). *p*‐Value was calculated using one‐way ANOVA with post‐hoc test. **, *p* < 0.01. J) RT‐qPCR showing the knockdown efficiency of FASN in HTC116 and HT29 cells. Error bars denote the s.d. (*n* = 3 per group). *p*‐Value was calculated using one‐way ANOVA with post‐hoc test. ***, *p* < 0.001. K,L) Representative histogram (K) and quantification (L) of flow cytometry with BODIPY staining showing lipid levels in the control, FASN knockdown (shFASN‐1 and shFASN‐2), *F. nucleatum*‐infected, and *F. nucleatum*‐infected with FASN knockdown (shFASN‐1 and shFASN‐2) HCT116 and HT29 cells. Error bars denote the s.d. (*n* = 3 per group). *p*‐Value was calculated using one‐way ANOVA with post‐hoc test. ***, *p* < 0.001. M,N) Representative histogram (M) and quantification (N) of flow cytometry with BODIPY staining showing lipid levels in the control, *F. nucleatum*‐infected, and *F. nucleatum*‐infected with cerulenin treatment (20 µm) HCT116 and HT29 cells. Error bars denote the s.d. (*n* = 3 per group). *p*‐Value was calculated using one‐way ANOVA with post‐hoc test. ***, *p* < 0.001.

We next examined the alteration of lipid accumulation in *F. nucleatum*‐infected non‐CCSCs. Before infection, we removed preexisting CCSCs by FACs (Figure S1E, Supporting Information). We observed that non‐CCSC lines, such as HCT116 and HT29, contain low levels of lipid droplets compared to those in CCSCs. Contrary to the observation in CCSCs, *F. nucleatum* infection significantly elevated lipid droplets in these cells (**Figure** **3**A–D). Transcriptome analysis showed that the expression of lipogenesis‐related genes was also elevated in *F. nucleatum‐*infected CRC cells (Figure 3E), among them, FASN was significantly upregulated (Figure 3F–I). FASN is a central enzyme involved in de novo lipogenesis, directly corresponding to triacylglycerol accumulation in lipid droplets.^[^ ^16^ ^]^ Functional investigation showed that FASN knockdown suppressed neutral lipid accumulation in *F. nucleatum*‐infected HTC116 and HT29 cells (Figure 3J–L). Consistently, FASN inhibitor, cerulenin, suppressed *F. nucleatum* infection‐elevated neutral lipid accumulation (Figure 3M,N). Together, the data show that *F. nucleatum* enhances lipid accumulation in non‐CCSCs by upregulating FASN expression.

### 2.6 *F. nucleatum* Promotes Non‐CCSCs to Gain Stem‐Like Features by Increasing Lipid Accumulation

**Figure 4:**
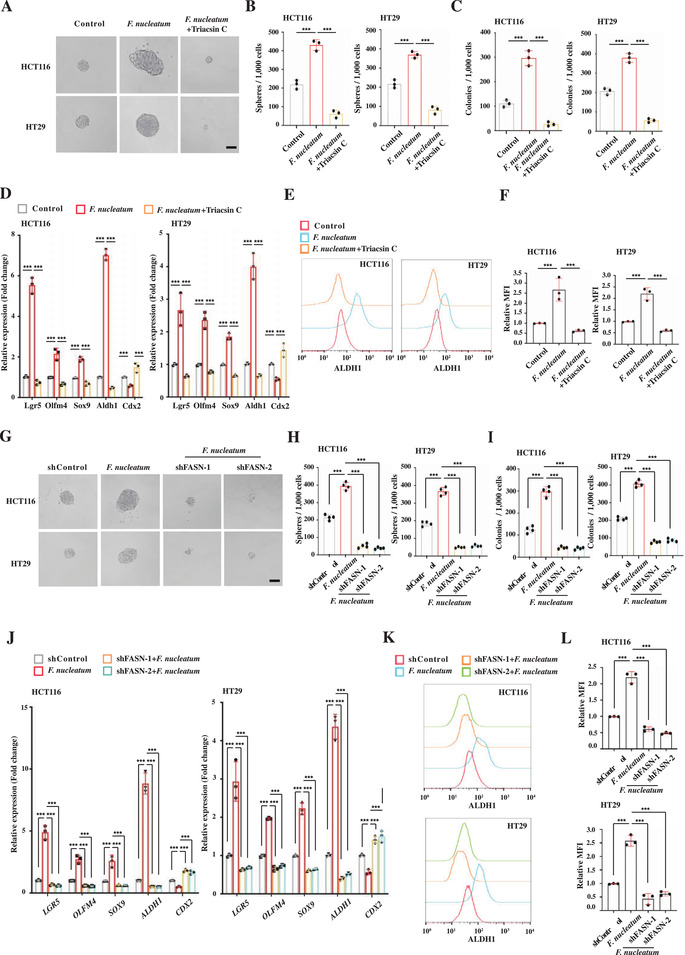
*F. nucleatum* promotes CRC cells to acquire stem‐like features by increasing lipid accumulation. A,B) Representative images (A) and quantification (B) of sphere formation of the control, *F. nucleatum*‐infected, and *F. nucleatum*‐infected with Triacsin C treatment (3 µm) HCT116 and HT29 cells. Error bars denote the s.d. (*n* = 3 per group). *p*‐Value was calculated using one‐way ANOVA with post‐hoc test. ***, *p* < 0.001. Scale bar, 100 µm. C) Clonogenicity assay of the control, *F. nucleatum*‐infected, and *F. nucleatum*‐infected with Triacsin C treatment (3 µm) HCT116 and HT29 cells. Error bars denote the s.d. (*n* = 3 per group). *p*‐Value was calculated using one‐way ANOVA with post‐hoc test. ***, *p* < 0.001. D) RT‐qPCR showing the relative expression levels of CCSC marker Lgr5, Olfm4, Sox9, and Aldh1 and differentiated marker Cdx2 in the control, *F. nucleatum*‐infected, and *F. nucleatum*‐infected with Triacsin C treatment (3 µm) HCT116 and HT29 cells. Error bars denote the s.d. (*n* = 3 per group). *p*‐Value was calculated using one‐way ANOVA with post‐hoc test. ***, *p* < 0.001. E,F) Representative histogram (E) and quantification (F) of flow cytometry showing ALDH1 expression in the control, *F. nucleatum*‐infected, and *F. nucleatum*‐infected with Triacsin C treatment (3 µm) HCT116 and HT29 cells. Error bars denote the s.d. (*n* = 3 per group). *p*‐Value was calculated using one‐way ANOVA with post‐hoc test. ***, *p* < 0.001. G,H) Representative images (G) and quantification (H) of sphere formation of the control, *F. nucleatum*‐infected, and *F. nucleatum*‐infected with FASN knockdown (shFASN‐1 and shFASN‐2) HCT116 and HT29 cells. Error bars denote the s.d. (*n* = 4 per group). *p*‐Value was calculated using one‐way ANOVA with post‐hoc test. ***, *p* < 0.001. Scale bar, 100 µm. I) Clonogenicity assay of the control, *F. nucleatum*‐infected, and *F. nucleatum*‐infected with FASN knockdown (shFASN‐1 and shFASN‐2) HCT116 and HT29 cells. Error bars denote the s.d. (*n* = 4 per group). *p*‐Value was determined using one‐way ANOVA with post‐hoc test. ***, *p* < 0.001. J) RT‐qPCR showing the relative expression levels of CCSC marker Lgr5, Olfm4, Sox9, and Aldh1 and differentiated marker Cdx2 in the control, *F. nucleatum*‐infected, and *F. nucleatum*‐infected with FASN knockdown (shFASN‐1 and shFASN‐2) HCT116 and HT29 cells. Error bars denote the s.d. (*n* = 3 per group). *p*‐Value was calculated using one‐way ANOVA with post‐hoc test. ***, *p* < 0.001. K,L) Representative histogram (K) and quantification (L) of flow cytometry showing ALDH1 expression in the control, *F. nucleatum*‐infected, and *F. nucleatum*‐infected with FASN knockdown (shFASN‐1 and shFASN‐2) HCT116 and HT29 cells. The red and blue histograms represent uninfected controls and infected cells, respectively. Error bars denote the s.d. (*n* = 3 per group). *p*‐Value was calculated using one‐way ANOVA with post‐hoc test. ***, *p* < 0.001.

To investigate whether increased lipid accumulation regulates gain of stem‐like features in the non‐CCSCs, we treated HCT116 and HT29 cells with Triacsin C, a selective acyl‐CoA synthetase inhibitor, inhibiting neutral lipid synthesis, and lipid droplet formation. We found that impaired neutral lipid accumulation by Triacsin C significantly suppressed *F. nucleatum*‐enhanced sphere‐formation, clonogenicity, and stem cell marker expression in HCT116 and HT29 cells (**Figure** **4**A–F). Consistently, FASN knockdown significantly suppressed *F. nucleatum*‐enhanced sphere‐formation, clonogenicity, and stem cell marker expression as well (Figure 4G–L). Together, the data show that lipid accumulation contributes to *F. nucleatum*‐mediated gain of stem‐like features in non‐CCSCs.

### 2.7 *F. nucleatum* Infection Activates Notch in Non‐CCSCs by Enhancing Numb Degradation

**Figure 5:**
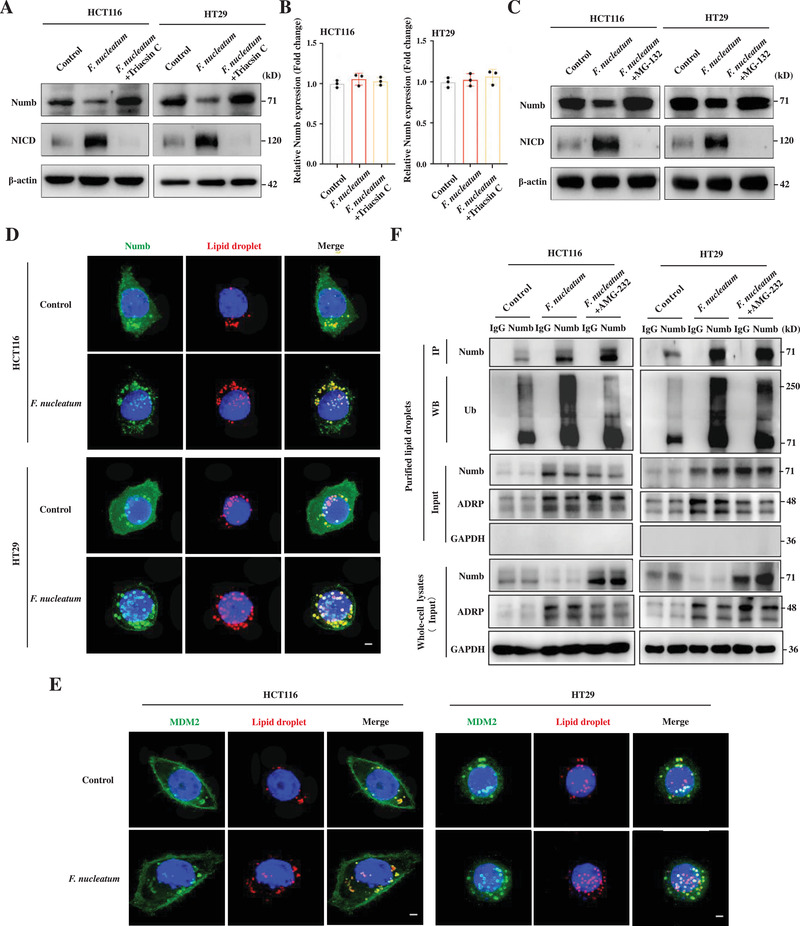
Lipid droplets promote Numb degradation by ubiquitination and activate Notch pathway in CRC cells. A,B) Western blot (A) and RT‐qPCR (B) showing Numb and NICD levels in the control and *F. nucleatum*‐infected HCT116 and HT29 cells. Triacsin C (3 µm) was used to inhibit lipid droplet accumulation. Error bars denote the s.d. (*n* = 3 per group). *p*‐Value was calculated using one‐way ANOVA with post‐hoc test. C) Western blot showing Numb and NICD levels in the control and *F. nucleatum*‐infected HCT116 and HT29 cells. MG‐132 (10 µm) was used to inhibit ubiquitination‐mediated protein degradation. D,E) Fluorescent staining (3D‐SIM) showing *F. nucleatum*‐elevated Numb (D) and MDM2 (E) localization on lipid droplets. Lipid droplets were visualized by Nile red staining. Scale bar, 5 µm. F) Immunoprecipitation and Western blot showing MDM2‐mediated Numb ubiquitination on the lipid droplets. Lipid droplets were purified from an equal number of control and *F. nucleatum*‐infected HCT116 and HT29 cells, followed by immunoprecipitation of Numb and Western blotting for Ubiquitin (Ub). AMG‐232 (4 µm) was used to inhibit MDM2 activity. ADRP and GAPDH were used as markers of lipid droplets and cytosolic fraction, respectively.

Notch signaling is known to play essential roles in cell reprograming and stemness maintenance.^[^ ^17^ ^]^ Since *F. nucleatum* infection promoted non‐CCSCs to gain stem‐like features (Figure 1), we hypothesized that *F. nucleatum* might manipulate Notch signaling in cells. To test this hypothesis, we examined Notch activity in HCT116 and HT29 cells infected with *F. nucleatum*. We observed that *F. nucleatum* infection significantly upregulated protein levels of the Notch intracellular domain (NICD), the active Notch form, while downregulating the protein level of the NICD negative regulator, Numb (**Figure** **5**A). When treating the cells with Triacsin C, we found that *F. nucleatum* infection failed to regulate the protein levels of NICD and Numb (Figure 5A). This observation suggested that *F. nucleatum* activated Notch signaling in non‐CCSCs and that the elevated Notch activity was regulated by *F. nucleatum*‐enhanced neutral lipid accumulation. Furthermore, we found that *F. nucleatum* infection only decreased Numb protein levels, while having no influence on Numb RNA levels (Figure 5B). Consistent with this observation, MG‐132, a potent proteinase inhibitor, abrogated *F. nucleatum* infection‐downregulated Numb and ‐upregulated NICD protein levels (Figure 5C). The data suggest that Notch activation is mediated by proteasomal degradation of Numb.

We next investigated the role of Notch signaling in *F. nucleatum*‐mediated gain of stem‐like features in the non‐CCSCs. We found that inhibition of Notch signaling by the *γ*‐secretase inhibitor DAPT significantly suppressed the capability of sphere and colony formation in *F. nucleatum*‐infected HCT116 and HT29 cells (Figure S4A–C, Supporting Information). Furthermore, knockdown of RBPJk, the major transcription effector of Notch signaling, suppressed xenograft tumor development in vivo (Figure S4D–G, Supporting Information). Together, the data show that *F. nucleatum* infection promotes Numb degradation, resulting in activation of Notch signaling and gain of CCSC features of CRC cells.

### 2.8 *F. nucleatum* Infection Enhances Numb Degradation by MDM2 on Lipid Droplets

Most mammalian cells store neutral lipids as lipid droplets for dynamic lipid homeostasis;^[^ ^18^ ^]^ however, emerging evidence suggests that lipid droplets can recruit ubiquitination machinery for protein degradation as well.^[^ ^7^ ^]^ Numb degradation was observed to be regulated by neutral lipid accumulation (Figure 5A–C). We therefore hypothesized that proteasomal degradation of Numb might be mediated by lipid droplet‐associated ubiquitination machinery. Super‐resolution imaging showed that Numb indeed localized on the lipid droplets along with MDM2, an E3 ubiquitin ligase well‐known for Numb ubiquitination (Figure 5D,E). In addition, *F. nucleatum* infection increased lipid droplet levels in the cells, which in turn recruited more Numb and MDM2 proteins to the lipid droplets (Figure 5D,E).

To investigate whether MDM2 ubiquitinates Numb on lipid droplets, we isolated lipid droplets from control and *F. nucleatum*‐infected cells using a well‐established approach.^[^ ^19^ ^]^ Adipose differentiation‐related protein (ADRP) and GAPDH were utilized as markers of lipid droplet and cytosolic fractions, respectively. We then examined ubiquitinated Numb levels in the lipid droplets by immunoprecipitation of Numb, followed by Western blotting for ubiquitin in equal amounts of control and *F. nucleatum*‐infected cells. We found that Numb ubiquitination indeed occurred on lipid droplets (Figure 5F). Furthermore, due to elevated lipid droplet levels in the *F. nucleatum*‐infected cells, Numb ubiquitination levels were significantly elevated as well (Figure 5F). In addition, we observed that Numb ubiquitination levels significantly decreased when the cells were pretreated with MDM2 inhibitor AMG‐232 (Figure 5F), suggesting that Numb ubiquitination on lipid droplets is mediated by MDM2.

### 2.9 MDM2 and VCP Are Recruited on Lipid Droplets by UBXD8

**Figure 6:**
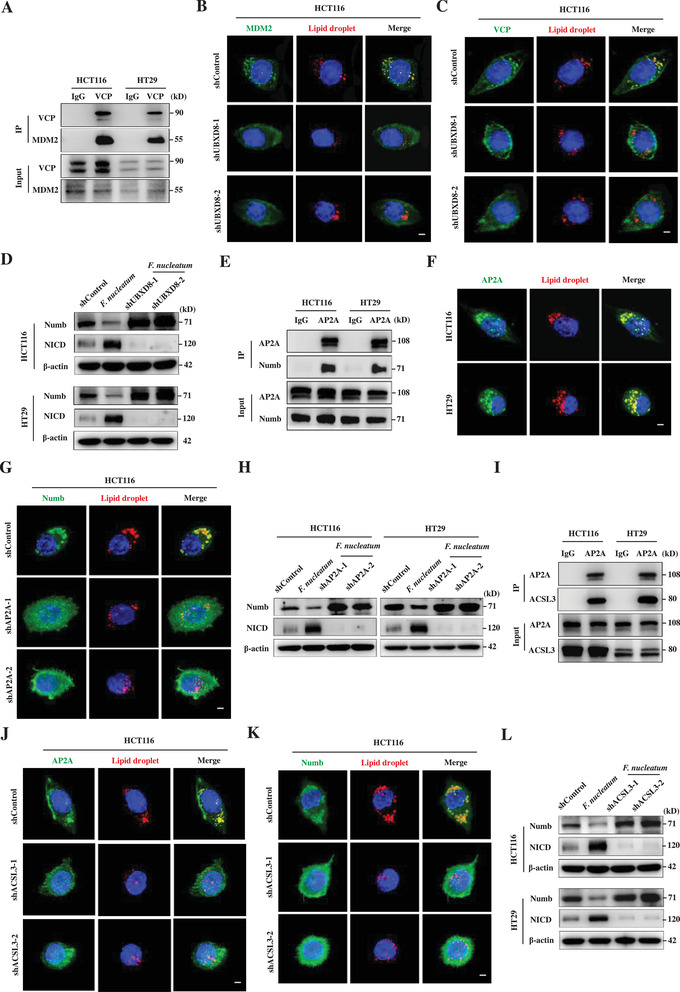
VCP, UBXD8, AP2A, and ACSL3 are required for lipid droplet‐mediated Numb ubiquitination. A) Immunoprecipitation and Western blot showing the interaction of VCP and MDM2 in HCT116 and HT29 cells. B,C) Fluorescent staining (3D‐SIM) showing the influence of UBXD8 knockdown (shUBXD8‐1 and shUBXD8‐2) on MDM2 (B) and VCP (C) localization on lipid droplets in *F. nucleatum*‐infected HCT116 cells. Scale bar, 5 µm. D) Western blot showing the influence of UBXD8 knockdown (shUBXD8‐1 and shUBXD8‐2) on Numb and NICD levels in HCT116 and HT29 cells. E) Immunoprecipitation and Western blot showing the interaction of AP2A and Numb in HCT116 and HT29 cells. F) Fluorescent staining (3D‐SIM) showing localization of AP2A on lipid droplets. Scale bar, 5 µm. G) Fluorescent staining (3D‐SIM) showing the influence of AP2A knockdown (shAP2A‐1 and shAP2A‐2) on Numb localization on lipid droplets in *F. nucleatum*‐infected HCT116 cells. Scale bar, 5 µm. H) Western blot showing the influence of AP2A knockdown (shAP2A‐1 and shAP2A‐2) on Numb and NICD levels in HCT116 and HT29 cells. I) Immunoprecipitation and Western blot showing the interaction of lipid protein ACSL3 and AP2A in HCT116 and HT29 cells. J,K) Fluorescent staining (3D‐SIM) showing the influence of ACSL3 knockdown (shACSL3‐1 and shACSL3‐2) on the localization of Numb (J) and AP2A (K) on lipid droplets in *F. nucleatum*‐infected HCT116 cells. Scale bar, 5 µm. L) Western blot showing the influence of ACSL3 knockdown (shACSL3‐1 and shACSL3‐2) on Numb and NICD levels in HCT116 and HT29 cells.

To investigate how MDM2 is recruited to the lipid droplets, we searched for potential MDM2‐interacting proteins in the Biological General Repository for Interaction Datasets (BioGRID), finding that valosin‐containing protein (VCP) potentially interacts with MDM2. VCP is an AAA+ ATPase, playing an important role in facilitating proteasome‐dependent degradation processes by helping release ubiquitin‐labelled proteins from membranes or interacting proteins.^[^ ^7^, ^20^ ^]^ Many reports have shown that VCP is recruited to lipid droplets by UBX domain‐containing protein 8 (UBXD8) and regulates lipid protein degradation.^[^ ^21^ ^]^ Here, immunoprecipitation confirmed that MDM2 does indeed interact with VCP (**Figure** **6**A). Super‐resolution imaging further showed that MDM2 and VCP were localized on lipid droplets, which were significantly suppressed when UBXD8 was knocked down (Figure 6B,C and Figure S5A–C, Supporting Information), indicating that recruitment of MDM2 and VCP onto lipid droplets is mediated by UBXD8. Furthermore, UBXD8 knockdown significantly suppressed *F. nucleatum* infection‐mediated Numb degradation and increased NICD levels (Figure 6D). Thus, UBXD8 recruits the VCP/MDM2 complex to lipid droplets, where MDM2 ubiquitinates Numb and VCP is likely to extract ubiquitin‐labeled Numb to proteasomes for degradation.

### 2.10 Numb Is Recruited to Lipid Droplets by AP2A and ACSL3

Although we did not discover lipid droplet proteins that interacted with Numb in the Biological General Repository for Interaction Datasets (BioGRID) database, we did find that adaptor protein complex 2 alpha subunit (AP2A) was potentially interacting with Numb. AP2 adaptor complex plays an important role in clathrin‐mediated endocytosis by interacting with plasma membrane lipids and proteins.^[^ ^22^ ^]^ Therefore, we hypothesized that AP2A might recruit Numb to the lipid droplets by interacting with lipid droplet membrane lipids or proteins. Immunoprecipitation confirmed that Numb and AP2A indeed interacted in HCT116 and HT29 cells (Figure 6E). Super‐resolution imaging further showed that AP2A was partially localized to lipid droplets in the *F. nucleatum*‐infected cells (Figure 6F). We then knocked down AP2A with two independent shRNAs (Figure S5D, Supporting Information), finding that knockdown of AP2A suppressed the recruitment of Numb to the lipid droplets, increased Numb protein level, and decreased NICD levels (Figure 6G,H and Figure S5E, Supporting Information). Thus, AP2A facilitates Numb recruitment to lipid droplets. To investigate how AP2A is recruited to the lipid droplets, we performed immunoprecipitation with an AP2A antibody. Mass spectrometry has shown that acyl‐CoA synthetase long‐chain 3 (ACSL3), a robust lipid droplet protein, potentially interacts with AP2A,^[^ ^23^ ^]^ which we further confirmed by Western blotting (Figure 6I and Figure S5F, Supporting Information). We then knocked down ACSL3 and examine how ACSL3 influences AP2A and Numb localization to lipid droplets (Figure S5G, Supporting Information). We observed that ACSL3 knockdown significantly suppressed AP2A and Numb localization to lipid droplets (Figure 6J,K and Figure S5H,I, Supporting Information), resulting in increased Numb protein levels and decreased Notch activity (Figure 6L). Together, the data suggest that AP2A likely acts as a cargo to recruit Numb to lipid droplets through ACSL3 for ubiquitination.

### 2.11 Lipid Droplet‐Mediated Numb Degradation Enhances Sphere Formation and Xenograft Growth

**Figure 7:**
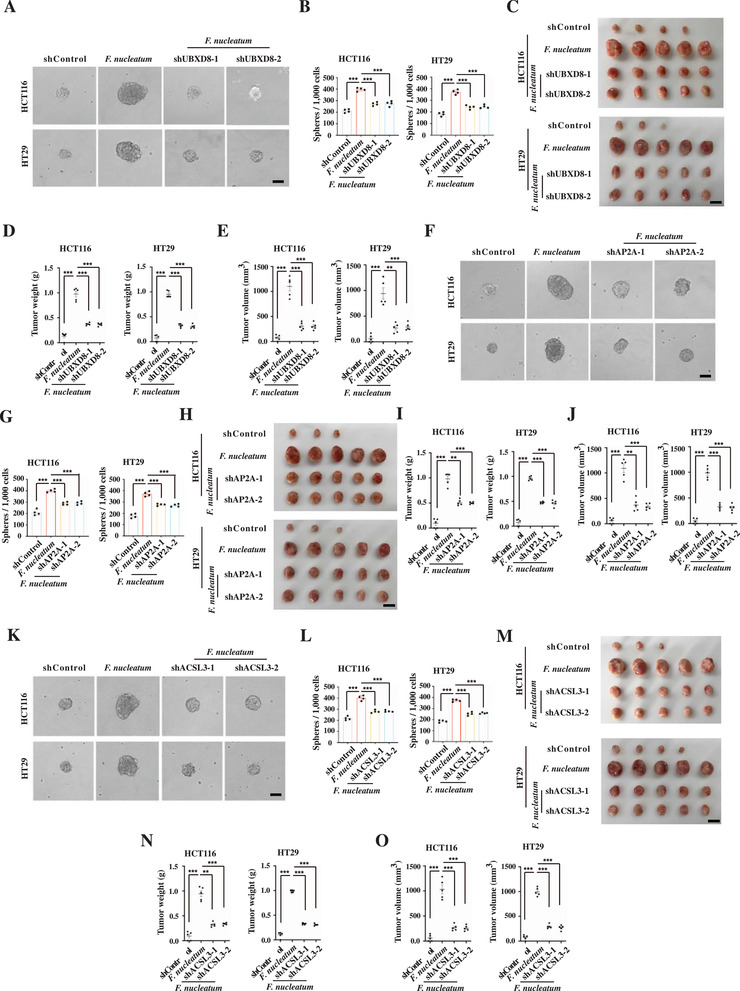
Lipid droplet‐mediated Numb degradation suppresses sphere formation and xenograft growth. A,B) Representative images (A) and quantification (B) of sphere formation of the control, *F. nucleatum*‐infected, and *F. nucleatum*‐infected with UBXD8 knockdown (shUBSD8‐1 and shUBXD8‐2) HCT116 and HT29 cells. Error bars denote the s.d. (*n* = 4 per group). *p*‐Value was calculated using one‐way ANOVA with post‐hoc test. ***, *p* < 0.001. Scale bar, 100 µm. C–E) Images (C), weight (D), and volume (E) of xenograft tumors developed from the control, *F. nucleatum*‐infected, and *F. nucleatum*‐infected with UBXD8 knockdown (shUBSD8‐1 and shUBXD8‐2) HCT116 and HT29 cells. Error bars denote s.e.m. (*n* = 5 per group). *p*‐Value was calculated using one‐way ANOVA with post‐hoc test. **, *p* < 0.01; ***, *p* < 0.001. F,G) Representative images (F) and quantification (G) of sphere formation of the control, *F. nucleatum*‐infected, and *F. nucleatum*‐infected with AP2A knockdown (shAP2A‐1 and shAP2A‐2) HCT116 and HT29 cells. Error bars denote the s.d. (*n* = 4 per group). *p*‐Value was calculated using one‐way ANOVA with post‐hoc test. ***, *p* < 0.001. Scale bar, 100 µm. H–J) Images (H), weight (I), and volume (J) of xenograft tumors developed from the control, *F. nucleatum*‐infected, and *F. nucleatum*‐infected with AP2A knockdown (shAP2A‐1 and shAP2A‐2) HCT116 and HT29 cells. Error bars denote s.e.m. (*n* = 5 per group). *p*‐Value was calculated using one‐way ANOVA with post‐hoc test. **, *p* < 0.01; ***, *p* < 0.001. K,L) Representative images (K) and quantification (L) of sphere formation of the control, *F. nucleatum*‐infected, and *F. nucleatum*‐infected with ACSL3 knockdown (shACSL3‐1 and shACSL3‐2) HCT116 and HT29 cells. Error bars denote the s.d. (*n* = 4 per group). *p*‐Value was calculated using one‐way ANOVA with post‐hoc test. ***, *p* < 0.001. Scale bar, 100 µm. M–O) Images (M), weight (N), and volume (O) of xenograft tumors developed from the control, *F. nucleatum*‐infected, and *F. nucleatum*‐infected with ACSL3 knockdown (shACSL3‐1 and shACLS3‐2) HCT116 and HT29 cells. Error bars denote s.e.m. (*n* = 5 per group). *p*‐Value was calculated using one‐way ANOVA with post‐hoc test. **, *p* < 0.01; ***, *p* < 0.001.

To investigate how lipid droplet‐mediated Numb degradation influences *F. nucleatum* infection‐enhanced self‐renewal and tumor progression, we performed a sphere formation assay and xenograft assay in HCT116 and HT29 cells when UBXD8, AP2A, or ACSL3 was knocked down. In cells, UBXD8 knockdown abrogated MDM2 recruitment to the lipid droplets, while AP2A or ACSL3 knockdown abrogated Numb recruitment to the lipid droplets, thus protecting Numb from degradation by lipid droplets. Furthermore, we found that knockdown of UBXD8, AP2A, or ACSL3 significantly suppressed sphere formation and xenograft growth (**Figure** **7**), indicating that lipid droplet‐mediated Numb degradation is important for *F. nucleatum*‐mediated gain of stem‐like features and tumor progression.

### 2.12 *F. nucleatum* Infection Regulates CPT1B and FASN through Activation of NF‐*κ*B

We next investigated how *F. nucleatum* infection regulated lipid accumulation through the expression of CPT1B and FASN. Some evidence shows that *F. nucleatum* activates NF‐*κ*B in CRC cells, while activated NF‐*κ*B has been reported to upregulate CPT1B.^[^ ^24^ ^]^ Thus, we hypothesized that *F. nucleatum* elevated CPT1B expression, likely through activation of NF‐*κ*B. Consistent with previous reports, we observed that *F. nucleatum* infection indeed activated NF‐*κ*B in CRC cells as well as CCSCs through the TLR4/MyD88 axis (Figure S6A, Supporting Information). In addition, inhibition of NF‐*κ*B by knockdown of TLR4, MYD88, or NF‐*κ*B suppressed *F. nucleatum*‐elevated CPT1B expression (Figure S6B–E, Supporting Information), indicating *F. nucleatum* upregulates CPT1B through activation of NF‐*κ*B. Interestingly, we found that NF‐*κ*B upregulated FASN in colorectal cells as well (Figure S6F, Supporting Information). In addition, inhibition of NF‐*κ*B suppressed *F. nucleatum* infection‐reduced lipid abundance in CCSCs and *F. nucleatum* infection‐increased lipid accumulation in HCT116 and HT29 CRC cells (Figure S6G,H, Supporting Information). Furthermore, inhibition of NF‐*κ*B significantly suppressed sphere‐formatting capability in CCSCs, HCT116, and HT29 cells (Figure S7A–D, Supporting Information). Together, the results suggest that *F. nucleatum* infection regulates the expression of CPT1B and FASN, which results in the alteration of lipid accumulation and stem‐like features in CCSCs and CRC cells.

## 3 Discussion

*F. nucleatum* has been reported to be abundant in the CRC microenvironment and an increased amount of *F. nucleatum* is associated with poor prognosis and recurrence.^[^ ^8^, ^9^ ^]^ Subsequent studies further show that *F. nucleatum* plays important roles in tumorigenesis and progression, which are mainly regulated by *F. nucleatum*‐mediated immune environment alteration, chemoresistance, and FadA adhesin.^[^ ^8^, ^9^ ^]^ However, it is unknown whether and how *F. nucleatum* manipulates the stemness of CCSCs, since CCSCs are responsible for CRC initiation and progression. In this study, we showed that *F. nucleatum* infects CCSCs and regular CRC cells, resulting in enhanced self‐renewal and proliferation of CCSCs and gain of stem‐like cell features in regular CRC cells. In addition, *F. nucleatum* regulated distinct lipid metabolisms in CCSCs and CRC cells. In CCSCs, *F. nucleatum* reduced lipid accumulation by promoting FAO, acquiring energy for CCSC self‐renewal and proliferation. In contrast, *F. nucleatum* increased lipid droplet levels in regular CRC cells by promoting fatty acid and triacylglycerol synthesis. Accumulated lipid droplets activate Notch signaling by recruiting Notch inhibitor Numb for degradation. Thus, we conclude that *F. nucleatum* promotes CRC progression by manipulating CRC stemness and reveal the novel role of lipid droplets in activating Notch signaling by mediating Numb degradation.

By acting as neutral lipid storage depots, lipid droplets are important for lipid metabolism, including storing excessive neutral lipids or metabolizing them for energy.^[^ ^7^, ^18^ ^]^ Lipid droplet homeostasis is associated with numerous diseases, including cancer progression.^[^ ^25^ ^]^ Recent studies show that lipid droplets also mediate lipid protein degradation by the ubiquitin‐proteasome for quality and quantity control.^[^ ^7^, ^18^ ^]^ In this study, we discovered that lipid droplets manipulate cell signaling by degrading Numb, an important non‐lipid droplet protein. Numb is a notable cell fate determinant, promoting stem cell differentiation by repressing Notch signaling, thus acting as a tumor suppressor in various cancers.^[^ ^26^ ^]^ It is known that Numb is ubiquitinated and degraded by MDM2,^[^ ^27^ ^]^ although where the ubiquitination occurs is not clear. In this study, we revealed that Numb is degraded by MDM2 on lipid droplets. We found that MDM2 was recruited to lipid droplets by AAA+ ATPase VCP and lipid droplet protein UBXD8. Furthermore, it is likely that MDM2 ubiquitinates Numb on lipid droplets, which is subsequently extracted by VCP for proteasome degradation. We also revealed that Numb is recruited to lipid droplets by adaptor protein AP2A and lipid droplet protein ACSL3. AP2A is known as a subunit of adaptor protein complex‐2, playing important roles in clathrin‐mediated endocytosis by interacting with the plasma membrane.^[^ ^22^ ^]^ Here we showed that AP2A is recruited to lipid droplets by ACSL3 and likely acts as a cargo protein to present Numb to MDM2 within lipid droplets.

NF‐*κ*B plays important roles in cancer progression by directly targeting a variety of genes or crosslinking to other signaling pathways.^[^ ^28^ ^]^ However, the response of NF‐*κ*B activation could be different in various cells, despite receiving the same stimulus.^[^ ^8^, ^29^ ^]^ We demonstrated that *F. nucleatum*‐activated NF‐*κ*B regulates distinct lipid metabolisms in CCSCs and regular CRC cells. In *F. nucleatum*‐infected CCSCs, NF‐*κ*B upregulates CPT1B expression, a key enzyme catalyzing the rate‐limiting step of FAO. In contrast, activated NF‐*κ*B upregulates FASN in regular CRC cells. CPT1B and FASN are likely indirectly regulated by NF‐*κ*B, since no NF‐*κ*B binding sites were identified in their promotors. In fact, CPT1B has been reported to be regulated by NF‐*κ*B through SUMO specific protease 2 (SENP‐2), while FASN is regulated by sterol regulatory element binding proteins (SREBPs), which are targets of NF‐*κ*B.^[^ ^24^, ^30^ ^]^ To this end, NF‐*κ*B regulates different metabolic genes in CCSCs and regular CRC cells, thereby mediating distinct lipid metabolisms.

Accumulation of lipid droplets in cancer cells is related to cancer progression and drug resistance.^[^ ^31^ ^]^ Therefore, despite the challenges, targeting lipid droplet biosynthesis has been considered cancer therapy.^[^ ^31^, ^32^ ^]^ Our work provides another lipid droplet‐related cancer therapeutic direction by suppressing recruitment of Numb or MDM2 to the lipid droplets. In addition, *F. nucleatum* abundance is positively associated with increased CCSC population in CRC tissue (Figure 1A) and CCSCs largely contribute to CRC initiation, metastasis, and chemoresistance.^[^ ^33^ ^]^ Thus, the measurement of *F. nucleatum* in the surgically removed tumor tissues could be an effective indicator to predict the outcome of CRC patients after treatments. Furthermore, chemotherapeutic drugs mainly kill differentiated cells, while having limited effects on CSCs, thus, frequently resulting in cancer recurrence due to CSC enrichment.^[^ ^34^ ^]^ Therefore, a combination of chemotherapy and anti‐*F. nucleatum* treatment may be an effective strategy for CRC patients with high level of *F. nucleatum*.

## 4 Experimental Section

### Mice and Xenograft Formation

Six‐week‐old male BALB/c nude mice were purchased from Weitonglihua Corporation (Beijing, China) and used in the studies. The mice were maintained in a specific‐pathogen free facility with a 12 h:12 h light:dark cycle at 21–23 °C, routinely checked by certified veterinarians, and deemed healthy prior to the tumor‐bearing experiments. After transplanting tumor cells, the mice were monitored daily for any signs of suffering or abnormal behavior. Mouse maintenance and procedures were approved by the Biomedical Research Ethics Committee of the Institute of Biophysics, Chinese Academy of Sciences. The experimental procedures were performed by following the relevant ethical regulations regarding animal research.

Xenograft formation was performed as described previously with slight modifications.^[^ ^8c^ ^]^ Briefly, the cells were co‐cultured with *F. nucleatum* at a multiplicity of infection (MOI) of 100 for 48 h. After washing with PBS three times, the cells were harvested by trypsinization and mixed with *F. nucleatum* at an MOI of 20. Next, 150 µL of the cells were subcutaneously injected into the flank of nude mice, while cells incubated with PBS were used as controls. Three hours after injection, the mice were intraperitoneally injected with 150 mg kg^−1^ piperacillin to kill bacteria.

### Cell and Bacterial Culture

HEK293T and CRC cell lines HCT116 and HT29 were obtained from American Type Culture Collection (ATCC). HCT116 and HT29 cells were cultured in RPMI‐1640 medium (GIBCO) supplemented with 10% FBS (PAN‐Biotech) and 1% Penicillin‐Streptomycin (HyClone). HEK293T cells were cultured in DMEM medium (GIBCO) supplemented with 10% FBS and 1% Penicillin‐Streptomycin. CCSCs were isolated and cultured as described previously.^[^ ^10^ ^]^ Briefly, CCSCs were cultured as spheres in ultralow‐attachment flasks (Corning) in DMEM/F12 (GIBCO) and supplemented with nonessential amino acids (Thermo Fisher), sodium pyruvate (Thermo Fisher), Penicillin‐Streptomycin (HyClone), N2 supplement (Invitrogen), B27 supplement (Invitrogen), 4 mg mL^−1^ heparin (Sigma‐Aldrich), 40 ng mL^−1^ epidermal growth factor (Invitrogen), and 20 ng mL^−1^ basic fibroblast growth factor (Invitrogen). To propagate in vitro, spheres were collected by gentle centrifugation, dissociated into single cells, and cultured for the formation of next generation spheres. All cell lines were kept at 37 °C and in a humidified 5% CO_2_ condition, and routinely tested for mycoplasma contamination by PCR.

*F. nucleatum* strain (ATCC 25 586) was obtained from China General Microbiological Culture Collection Center and cultured at 37 °C under anaerobic condition in brain heart infusion (BHI) broth.

### shRNA Lentivirus Preparation and Infection

shRNAs were synthesized (Sangon Biotech) and cloned into the lentiviral vector pLKO.1 (Addgene). The lentiviral constructs were co‐transfected with the psPAX2 packaging plasmid and the pMD2.G envelope plasmid into HEK293T cells. The viruses were collected 48 h after transfection and used to infect the cells. shRNA sequences are shown in Table S1, Supporting Information.

### Patient Specimen

The CRC specimens used for measuring *F. nucleatum* abundance and preparing organoids were obtained from the 7th medical center of PLA general hospital with informed consent from all donors. All studies were approved by the Ethics Committee of the 7th Medical Center of PLA General Hospital and the Institute of Biophysics, Chinese Academy of Sciences.

### RNA‐seq

Total RNA was isolated using TRIzol reagent (Invitrogen) and rRNA was removed using Ribo‐Zero Gold rRNA Removal Kit (Illumina). The RNA libraries were prepared using NEB Next Ultra II Directional RNA Library Prep Kit for Illumina (NEB) according to the manufacturer's instructions. Three independent biological replicates were performed for RNA‐seq. FastQC package was used to assess the sequencing quality of raw data and the low‐quality reads were filtered by Trim Galore; the processed data were mapped to the human reference genome (hg38) using STAR Aligner (v2.7.3a). Count files of the aligned reads were generated by the HTSeq‐count tool. Differential expression analysis was performed by DESeq2 packages. Gene ontology enrichment was analyzed by R package clusterProfiler.

### RT‐qPCR

Total RNA was isolated using TRIzol (Invitrogen) and evaluated by agarose gel electrophoresis and the absorbance ratio of 260/280 and 260/230. The cDNA was synthesized using a HiScript II qRT SuperMix (Vazyme) and potential contaminant DNA was removed by adding DNase. Realtime PCR was performed on the QuantStudio 3 real‐time PCR instrument using the primers shown in Table S1, Supporting Information. All primers were purchased from Sangon Biotech and validated for specificity by amplifying serial dilution of template cDNA. The expression of each gene was defined from the threshold cycle (Ct) and the relative levels were calculated using the 2‐ΔΔCt method and normalized to the levels of GAPDH.

### Immunofluorescence

For staining *F. nucleatum* and ALDH1 in the CRC tissues, anti‐*F. nucleatum* antibody was prepared as described previously.^[^ ^35^ ^]^ The antibody was validated for specificity by immunofluorescence. CRC specimens were embedded in O.C.T. and cut into 7 µm‐thick sections. The sections were stained with anti‐ALDH1 and anti‐*F. nucleatum* antibody, followed by goat anti‐rabbit IgG antibody.

For evaluating lipid droplet‐mediated Numb degradation, the cells were plated on a culture slide (BD Biosciences). After being fixed in 4% PFA and permeabilized in 0.5% Triton X‐100/PBS, the cells were blocked in 5% normal goat serum for 1 h at room temperature and incubated with primary antibodies overnight at 4 °C. The cells were then stained with 488‐labeled secondary antibody (Life Technologies) for 1 h at room temperature. For lipids or lipid droplets staining, the cells were incubated with Nile red (1 µm) for 30 min at 37 °C. The slides were observed under the DeltaVision OMX V3 imaging system (Cytiva, GE Healthcare) or a Confocal FV1200 (OLYMPUS). The 3D‐SIM images were reconstructed using a maximum intensity projection of three dimensions. Antibody information including source, catalog number, and dilution is shown in Table S2, Supporting Information.

### Immunoprecipitation Assay

CRC cells or purified lipid droplets were lysed in RIPA buffer (150 mm NaCl, 0.5% sodium deoxycholate, 0.1% SDS, 1% NP‐40, 1 mm EDTA, and 50 mm Tris, pH 8.0, containing protease inhibitor cocktail) for 30 min at 4 °C. After centrifugation at 12 000 *g* for 30 min, the supernatants were pre‐cleared with protein A/G beads (Thermo scientific) for 1 h followed by incubating with primary antibodies overnight at 4 °C. New protein A/G beads were then added for immunoprecipitation. Precipitates were collected and detected by mass spectrometry or Western blotting with the indicated antibodies.

### Western Blot

Whole cell lysates were prepared in RIPA buffer at 4 °C for 30 min, followed by separation with 10% SDS‐PAGE. Proteins were then transferred to a Hybond membrane (Amersham). The membranes were blocked in 5% fat‐free milk and incubated with primary antibodies overnight at 4 °C, and then probed for 1 h with secondary horseradish peroxidase (HRP)‐conjugated anti‐mouse or anti‐rabbit IgG. After extensive washing with PBST, the target proteins were detected using enhanced chemiluminescence according to manufacturer's instructions. *β*‐actin was used as the internal control. Antibody information is shown in Table S2, Supporting Information.

### Sphere Formation Assay

To measure tumor sphere formation, cells were incubated with *F. nucleatum* at an MOI of 100 for 4 days; PBS treated cells were set as the negative control. Cells were plated in 24‐well ultra‐low attachment plates (Corning) at 1000 cells/well in the above‐mentioned CCSC culture medium. After 2 weeks, tumor spheres were counted using an inverted microscope (Olympus).

### Clonogenicity Assay

Cells were incubated with *F. nucleatum* at an MOI of 100 for 4 days. Cells were plated at 1000 cells/well in 12‐well plate with 10% FBS‐containing culture medium for growth for 1 week. The colonies were stained with crystal violet and counted; a colony was defined as having >50 cells.

### Flow Cytometry

For examining CCSCs, single‐cells were incubated with PE‐conjugated anti‐CD133 and APC‐conjugated anti‐CD44. Alternatively, ALDH1 levels were analyzed using the Aldeflour kit (STEMCELL). For examining lipids, single cells were incubated with BODIPY (1 µm) for 30 min at 37 °C. The samples were analyzed using FACScalibur (BD Biosciences), and data were analyzed using FlowJo software.

### Fatty Acid Oxidation and ATP Production Assay

Cells were incubated with [9,10‐^3^H(N)]‐Oleic acid (20 µm, 1 µCi/well; PerkinElmer) for 2 h at 37 °C in a humidified 5% CO_2_ atmosphere. The FAO rate was determined by measuring ^3^H_2_O production. ATP Assay Kit (Solarbio) was used to detect ATP production according to the manufacturer's instruction.

### Lipid Droplets Isolation

Lipid droplet isolation was performed following the protocol described previously.^[^ ^19^ ^]^ Briefly, after washing with PBS three times, the cells were scraped off with 1 mL Buffer A (20 mm tricine, 250 mm sucrose, pH 7.8, and 0.5 mm PMSF) and put into a 15 mL centrifuge tube, followed by centrifugation at 1000 *g* for 3 min at 4 °C. The cell pellets were then resuspended with 10 mL Buffer A, incubated on ice for 20 min, and disrupted using a nitrogen bomb at a pressure of 700 psi for 15 min on ice. The supernatant cell lysates were collected into to new 15 mL centrifuge tube by centrifugation at 1000 *g* for 3 min at 4 °C. After carefully placing 2 mL Buffer B (20 mm HEPES, 100 mm KCl, and 2 mm MgCl_2_, pH 7.4) on the top, the top layer of white material (lipid droplets) was collected by centrifugation at 15 000 *g* for 30 min at 4 °C.

### Tumor Organoids Culture

CRC organoids were cultured as previously described.^[^ ^36^ ^]^ To evaluate the influence of *F. nucleatum* on CRC organoid formation, CRC tissues were dissociated and co‐cultured at an MOI of 100 *F. nucleatum* under CRC organoids culture condition. The *F. nucleatum*‐infected and ‐control organoids were further dissociated into single cells and 1000 cells of each condition were reseeded to grow into organoids. The organoid‐formatting capability was evaluated by measuring organoids size and number.

### Statistical Analysis

Statistical analysis was performed using the GraphPad Prism software (version 5) using Student's *t*‐tests, one‐way ANOVA, two‐way ANOVA, or Pearson correlation analysis. All graphs show mean ± s.d. or mean ± s.e.m. as indicated in the figure legends. *p*‐Values of less than 0.05 were considered statistically significant. Each experiment was conducted with biological replicates and repeated a minimum of three times. Mice were randomly allocated to experimental groups.

## Conflict of Interest

The authors declare no conflict of interest.

## Author Contributions

H.L. and J.D. contributed equally to this work. H.L. and P.B. came up with the concept, designed the experiments, and wrote the manuscript. H.L. performed most experiments with assistance from S.C. for *F. nucleatum* strain purchase and culture, S.L. for 3D‐SIM imaging, H.Z. for fatty acid oxidation rate detection. J.D., H.C., and G.C. provided the patient samples. P.L. assisted the [9,10‐^3^H(N)]‐Oleic acid preparation and lipid droplet isolation.

## Supporting information

Supporting Information

## Data Availability

Research data are not shared.
