# Supplementary material for: Fusobacterium nucleatum Promotes Colorectal Cancer Cell to Acquire Stem Cell‐Like Features by Manipulating Lipid Droplet‐Mediated Numb Degradation
Source: Adv Sci (Weinh). 2022 Feb 15;9(12):2105222. doi: 10.1002/advs.202105222 (PMC9035998; doi:10.1002/advs.202105222)
Supplement: Supplementary file 1 — Supporting Information [file ADVS-9-2105222-s001.pdf]

ADVANCED SCIENCE
Open Access

Supporting Information

for *Adv. Sci.*, DOI 10.1002/advs.202105222

Fusobacterium nucleatum Promotes Colorectal Cancer Cell to Acquire Stem Cell-Like Features by Manipulating Lipid Droplet-Mediated Numb Degradation

*Haiyang Liu, Junfeng Du, Shanshan Chao, Shuoguo Li, Huiyun Cai, Hongjie Zhang, Gang Chen, Pingsheng Liu and Pengcheng Bu**

ADVANCED SCIENCE
Open Access

Supporting Information

for *Adv. Sci.*, DOI: 10.1002/advs.202105222

Fusobacterium nucleatum promotes colorectal cancer cell to acquire stem cell-like features by manipulating lipid droplet-mediated Numb degradation

Haiyang Liu[1], Junfeng Du[2,3,4], Shanshan Chao[1,5], Shuoguo Li[6], Huiyun Cai[2], Hongjie Zhang[7], Gang Chen[2,4], Pingsheng Liu[8,9], Pengcheng Bu[1,5,9,10]*

***Fusobacterium nucleatum* promotes colorectal cancer cell to acquire stem cell-like features by manipulating lipid droplet-mediated Numb degradation**

Haiyang Liu[1#], Junfeng Du[2,3,4#], Shanshan Chao[1,5], Shuoguo Li[6], Huiyun Cai[2], Hongjie Zhang[7], Gang Chen[2,4], Pingsheng Liu[8,9], Pengcheng Bu[1,5,9,10*]

[1] Key Laboratory of RNA Biology, Key Laboratory of Protein and Peptide Pharmaceutical, Institute of Biophysics, Chinese Academy of Sciences, Beijing 100101, China.

[2] Department of General Surgery, the 7th Medical Center, Chinese PLA General Hospital, Beijing 100700, China.

[3] The 2nd School of Clinical Medicine, Southern Medical University, Guangdong 510515, China.

[4] Medical Department of General Surgery, the 1st Medical Center, Chinese PLA General Hospital, Beijing 100853, China.

[5] College of Life Sciences, University of Chinese Academy of Sciences, Beijing 100049, China.

[6] Center for Biological Imaging, Institute of Biophysics, Chinese Academy of Sciences, Beijing, 100101, China.

[7] The core facility, Institute of Biophysics, Chinese Academy of Sciences, Beijing, 100101, China.

[8] National Laboratory of Biomacromolecules, Institute of Biophysics, Chinese Academy of Sciences, Beijing,100101, China

[9] Center for Excellence in Biomacromolecules, Chinese Academy of Sciences, Beijing 100101, China.

[10] Lead contact

* Correspondence: Pengcheng Bu (bupc@ibp.ac.cn)

# H.Y. Liu and J.F.Du contributed equally to this work

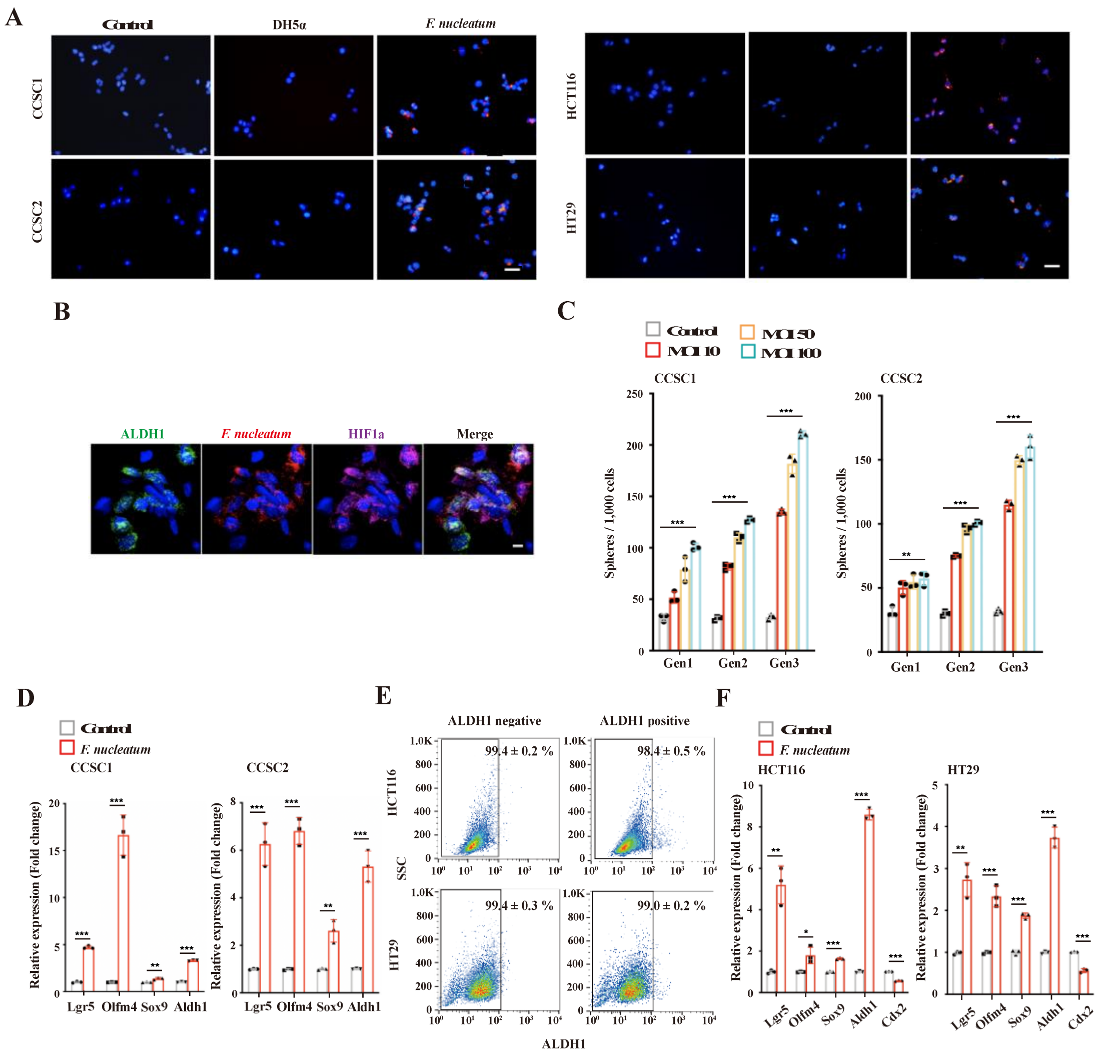

Figure S1. *F. nucleatum* promotes CCSC sphere formation.

(A) Fluorescent staining showing *F. nucleatum* infection of the cells. Uninfected (Control), DH5α infected, and *F. nucleatum*-infected CCSCs, HCT116, and HT29

cells were stained with the anti-*F. nucleatum* antibody.

(B) Fluorescent staining (3D-SIM) showing the association of hypoxia and ALDH1 positive cells infected with *F. nucleatum* in the CRC tissues. The representative images were showed as ALDH1 positive cell-enriched areas.

(C) CCSC sphere formation during serial passages after *F. nucleatum* infection at different MOIs. Error bars denote the s.d. (n = 3 per group). Scale bar, 50 μm. *P*-value was calculated using Two-way ANOVA. **, $p < 0.01$; ***, $p < 0.001$.

(D) RT-qPCR showing the relative expression levels of the CCSC markers in the control and *F. nucleatum*-infected CCSC sphere cells. Error bars denote the s.d. (n = 3 per group). *P*-value was calculated using one-way ANOVA with post-hoc test. **, $p<0.01$; ***, $p < 0.001$.

(E) Representative flow cytometry plots showing how pre-existed ALDH1+ cells were removed from HTC116 and HT29 cells.

(F) RT-qPCR showing the relative expression levels of CCSC markers Lgr5, Olfm4, Sox9, and Aldh1, and differentiated marker Cdx2 in the control and *F. nucleatum*-infected HCT116 and HT29 cells. Error bars denote the s.d. (n = 3 per group). *P*-value was calculated using one-way ANOVA with post-hoc test. *, $p < 0.05$; **, $p < 0.01$; ***, $p < 0.001$.

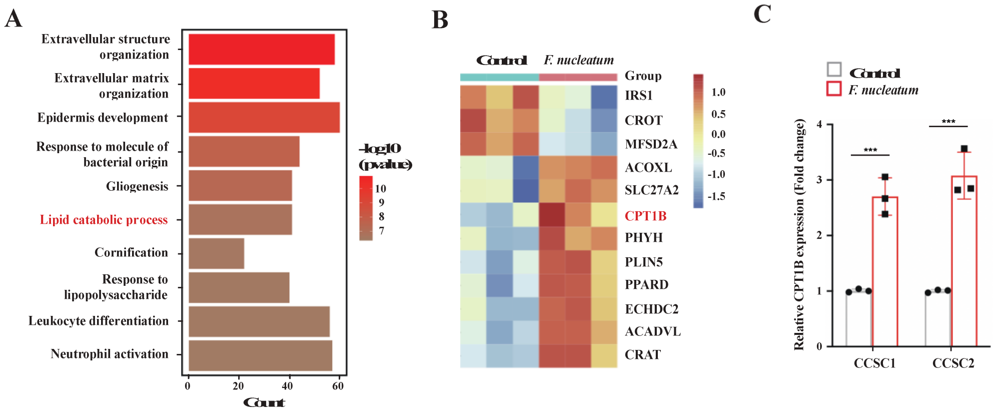

Figure S2. *F. nucleatum* infection upregulates fatty oxidation-associated gene expression in CCSCs.

(A) Gene ontology analyses on the RNA-seq dataset showing the differentially

expressed genes in the control and *F. nucleatum*-infected CCSCs.

(B) Heatmap of the RNA-seq dataset showing the differentially expressed genes in the control and *F. nucleatum* infected CCSCs.

(C) RT-qPCR showing CPT1B expression in the control and *F. nucleatum*-infected CCSC sphere cells. Error bars denote s.d. (n = 3 per group). *P*-value was calculated using one-way ANOVA with post-hoc test. ***, $p < 0.001$.

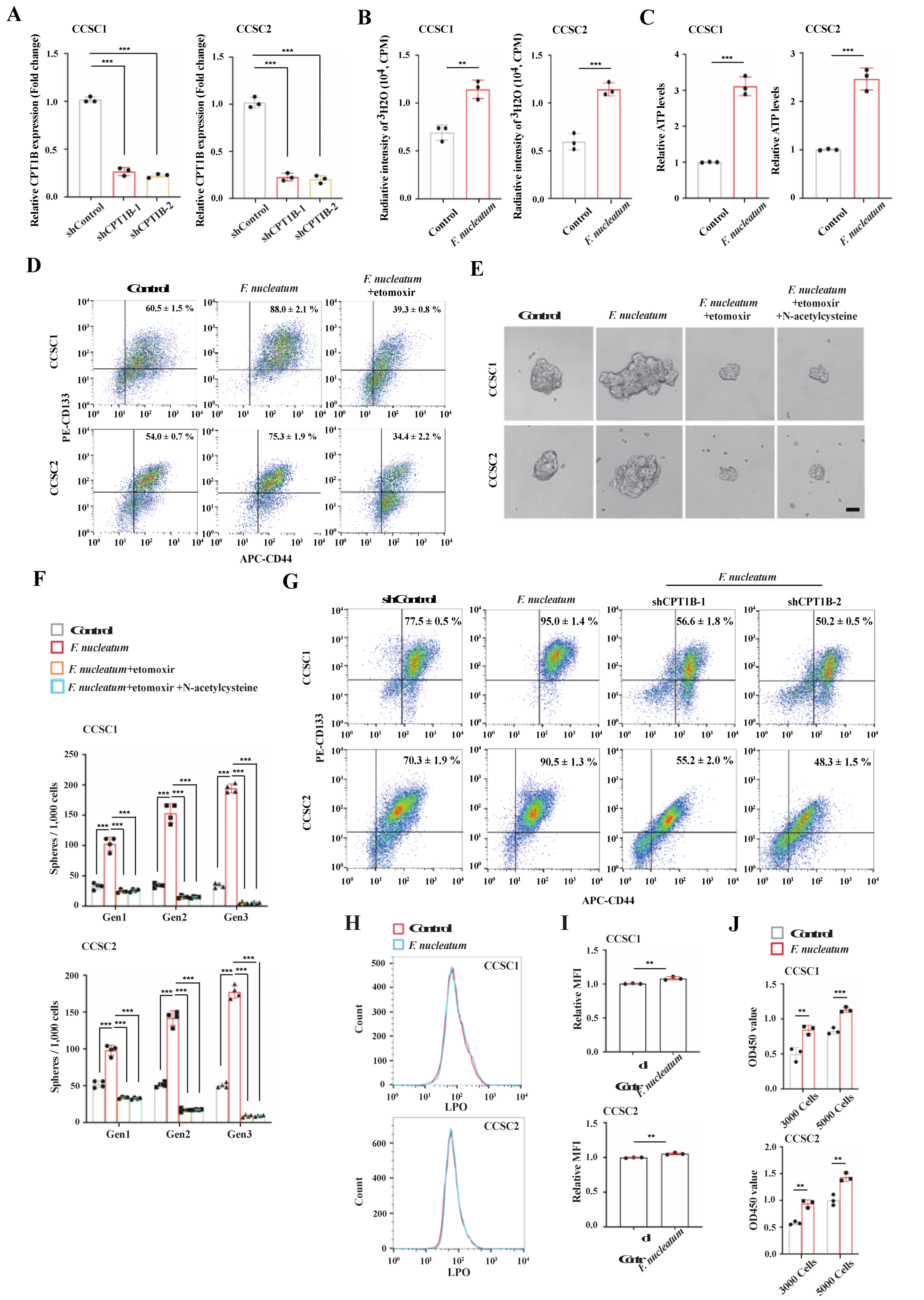

Figure S3. *F. nucleatum* promotes CCSC self-renewal by enhancing CPT1B-mediated fatty acid oxidation.

(A) RT-qPCR showing the knockdown efficiency of CPT1B. Error bars denote the

s.d. (n = 3 per group). *P*-value was calculated using one-way ANOVA with post-hoc test. ***, $p < 0.001$.

(B and C) $^{3}H2O$ radioactivity (B) and ATP levels (C) indicating fatty acid oxidation rate in the control and *F. nucleatum*-infected CCSC sphere cells. Error bars denote the s.d. (n = 3 per group). *P*-value was calculated using Student's t-tests. ***, $p < 0.001$.

(D) Flow cytometry showing the percentage of $CD133^{+}CD44^{+}$ CCSCs in control, *F. nucleatum*-infected, and *F. nucleatum*-infected with etomoxir treatment (50 μM) CCSC sphere cells.

(E and F) Representative images (E) and quantification (F) of sphere formation of the control, *F. nucleatum*-infected, *F. nucleatum*-infected with etomoxir treatment (50 μM), and *F. nucleatum*-infected with etomoxir treatment (50 μM) and N-acetylcysteine (5 nM) CCSC sphere cells. Error bars denote the s.d. (n = 4 per group). *P*-value was calculated using one-way ANOVA with post-hoc test. ***, $p < 0.001$. Scale bar, 100 μm

(G) Flow cytometry showing $CD133^{+}CD44^{+}$ CCSCs in the control, *F. nucleatum*-infected, and *F. nucleatum*-infected with CPT1B knockdown (shCPT1B-1 and shCPT1B-2) CCSC sphere cells.

(H and I) Representative histogram (H) and quantification (I) of flow cytometry showing lipid peroxidation (LPO) levels in the control and *F. nucleatum*-infected, CCSCs. Error bars denote the s.d. (n = 3 per group). *P*-value was calculated using Student's t-tests. **, $p < 0.01$.

(J) CCK8 assay showing the effect of *F. nucleatum* infection on CCSC viability. Error bars denote the s.d. (n = 3 per group). *P*-value was calculated using one-way ANOVA with post-hoc test. **, $p < 0.01$; ***, $p < 0.001$.

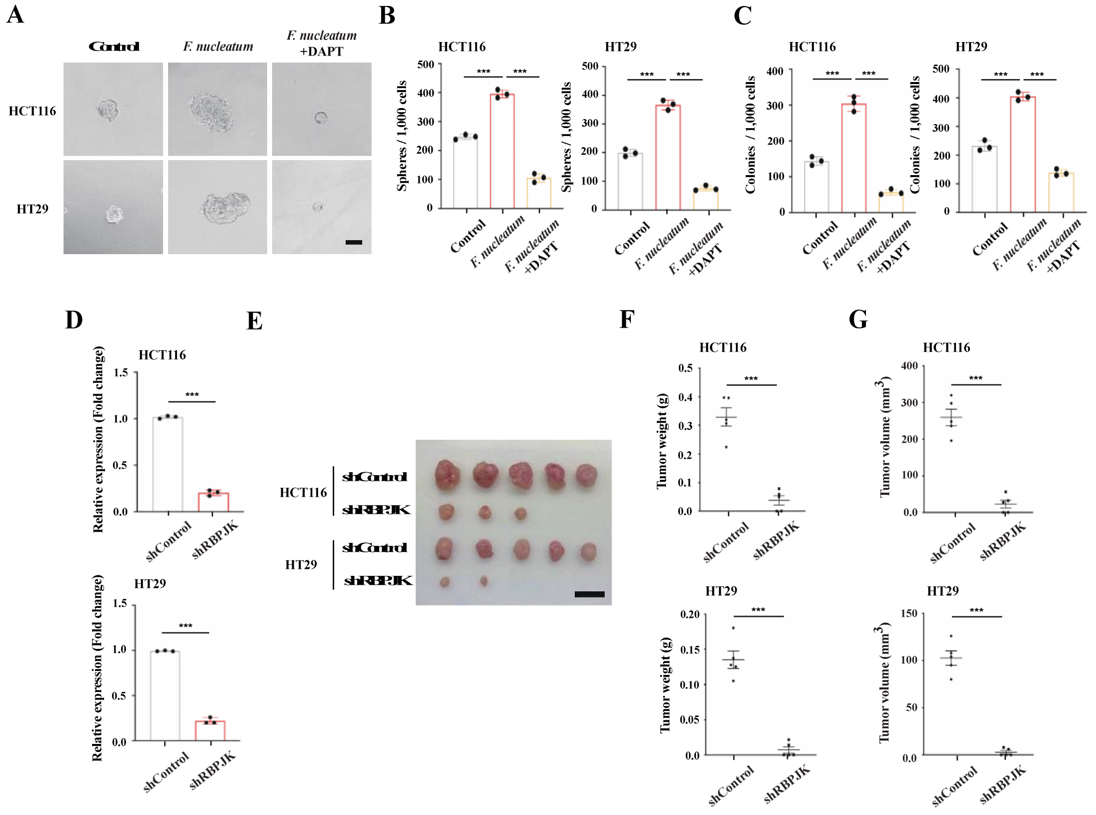

Figure S4. ***F. nucleatum*****-activated Notch signaling promotes non-CCSCs to acquire stem-like features.**

(A and B) Representative images (A) and quantification (B) of sphere formation of

the control, *F. nucleatum*-infected, and *F. nucleatum*-infected with DAPT treatment (10 μM) HCT116 and HT29 cells. DAPT was used to inhibit Notch signaling. Error bars denote the s.d. (n = 3 per group). *P*-value was calculated using one-way ANOVA with post-hoc test. ***, $p < 0.001$. Scale bar, 100 μm.

(C) Clonogenicity assay of the control, *F. nucleatum*-infected, and *F. nucleatum*-infected with DAPT treatment HCT116 and HT29 cells. Error bars denote the s.d. (n = 3 per group). *P*-value was determined using one-way ANOVA with post-hoc test. ***, $p < 0.001$.

(D) RT-qPCR showing the knockdown efficiency of RBPJk. Error bars denote the s.d. (n = 3 per group). *P*-value was determined using Student's t-test. ***, $p < 0.001$.

(E-G) Images (E), weight (F) and volume (G) of xenograft tumors developed from the *F. nucleatum*-infected control and RBPJk-knockdown HCT116 and HT29 cells. Error bars denote the s.e.m. (n = 5 per group). *P*-value was calculated using Student's t-test. ***, $p < 0.001$. Scale bar, 1 cm.

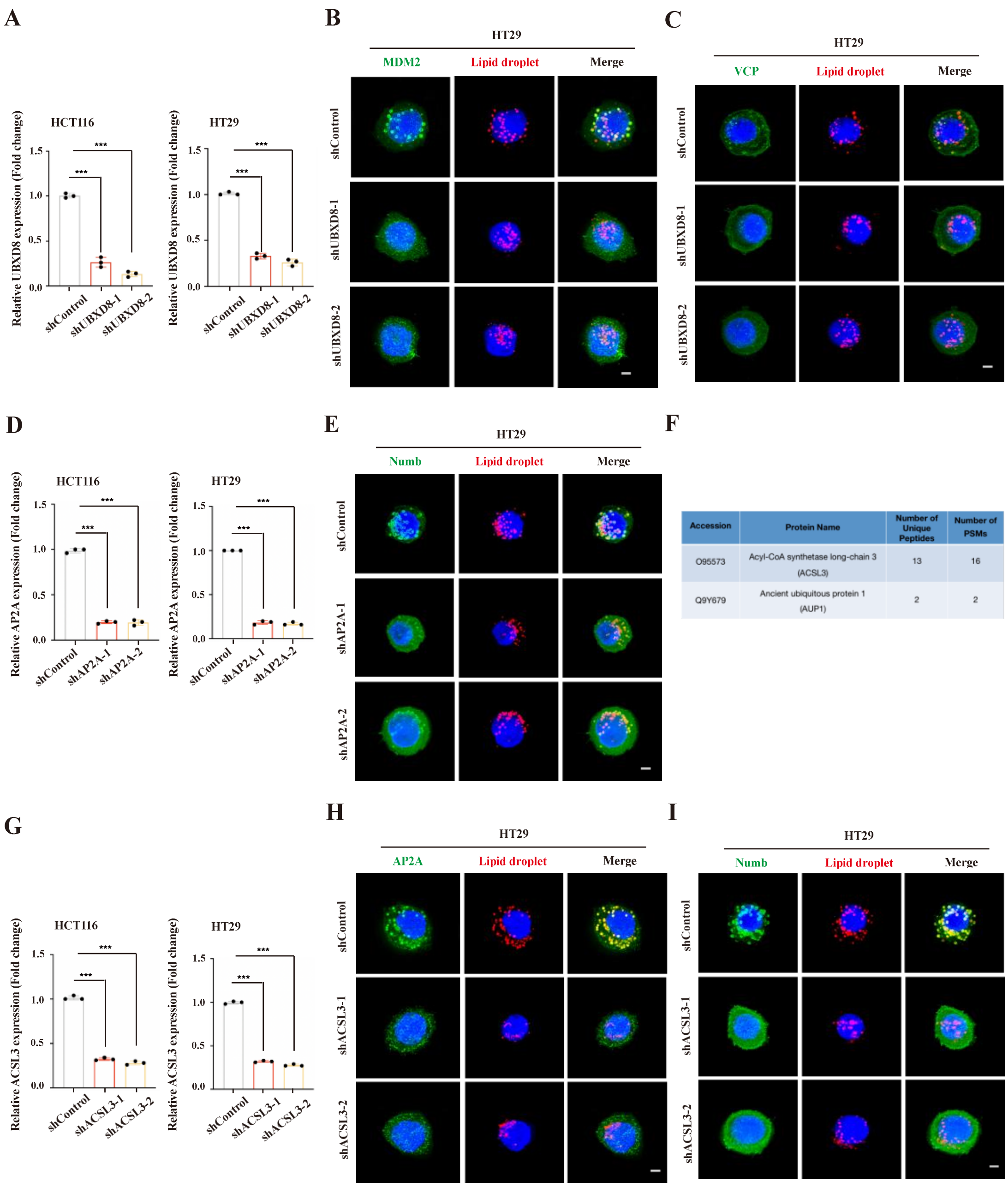

Accession	Protein Name	Number of Unique Peptides	Number of PSMs
O95573	Acyl-CoA synthetase long-chain 3 (ACSL3)	13	16
Q9Y679	Ancient ubiquitous protein 1 (AUP1)	2	2

Figure S5. Recruitment of MDM2 and Numb on lipid droplets.

(A) RT-qPCR showing the knockdown efficiency of UBXD8 in HTC116 and HT29 cells. Error bars denote the s.d. (n = 3 per group). *P*-value was calculated using one-

way ANOVA with post-hoc test. ***, $p < 0.001$.

(B and C) Fluorescent staining (3D-SIM) showing the influence of UBXD8 knockdown (shUBXD8-1 and shUBXD8-2) on the location of MDM2 (B) and VCP (C) on lipid droplets in *F. nucleatum*-infected HT29 cells.

(D) RT-qPCR showing the knockdown efficiency of AP2A in HTC116 and HT29 cells. Error bars denote the s.d. (n = 3 per group). *P*-value was calculated using one-way ANOVA with post-hoc test. ***, $p < 0.001$.

(E) Fluorescent staining (3D-SIM) showing the influence of AP2A knockdown (shAP2A-1 and shAP2A-2) on Numb localization on lipid droplets in *F. nucleatum*-infected HT29 cells.

(F) Mass spectrometry analysis of AP2A potentially interacting lipid droplet protein ACSL3.

(G) RT-qPCR showing the knockdown efficiency of ACSL3 in HTC116 and HT29 cells. Error bars denote the s.d. (n = 3 per group). *P*-value was calculated using one-way ANOVA with post-hoc test. ***, $p < 0.001$.

(H–I) Fluorescent staining (3D-SIM) showing the influence of ACSL3 knockdown (shACSL3-1 and shACSL3-2) on AP2A (H) and Numb (I) localization on lipid droplets in *F. nucleatum*-infected HT29 cells. Scale bar, 5 μm. ***, $p < 0.001$.

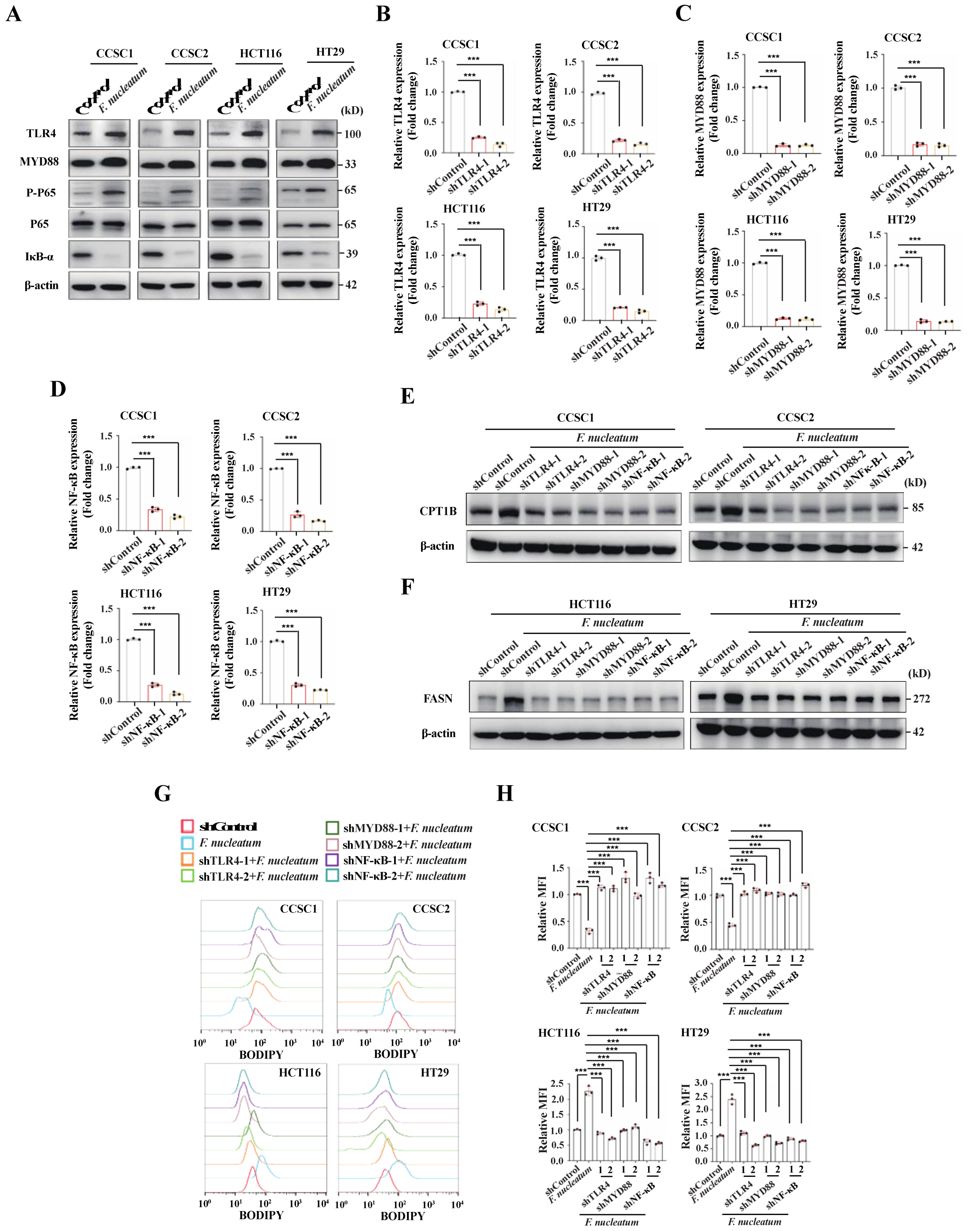

Figure S6. *F. nucleatum* upregulates CPT1B and FASN through NF-κB pathway.

(A) Western blot showing *F. nucleatum* activates NF-κB in CCSCs, HCT116, and HT29 cells.

(B-D) RT-qPCR showing knockdown efficiency of TLR4 (B), MYD88 (C), and NF-κB (D). Error bars denote the s.d. (n = 3 per group). *P*-values were determined using one-way ANOVA with post-hoc test. ***, $p < 0.001$.

(E and F) Western blot showing inhibition of NF-κB suppressed CPT1B expression in *F. nucleatum*-infected CCSCs (E) and FASN expression in *F. nucleatum*-infected HCT116 and HT29 cells (F).

(G and H) Representative histogram (G) and quantification (H) of flow cytometry with BODIPY staining showing the influence of NF-κB inhibition on lipid content in *F. nucleatum*-infected CCSCs, HCT116, and HT29 cells. Error bars denote the s.d. (n = 3 per group). *P*-values were determined using one-way ANOVA with post-hoc test. ***, $p < 0.001$.

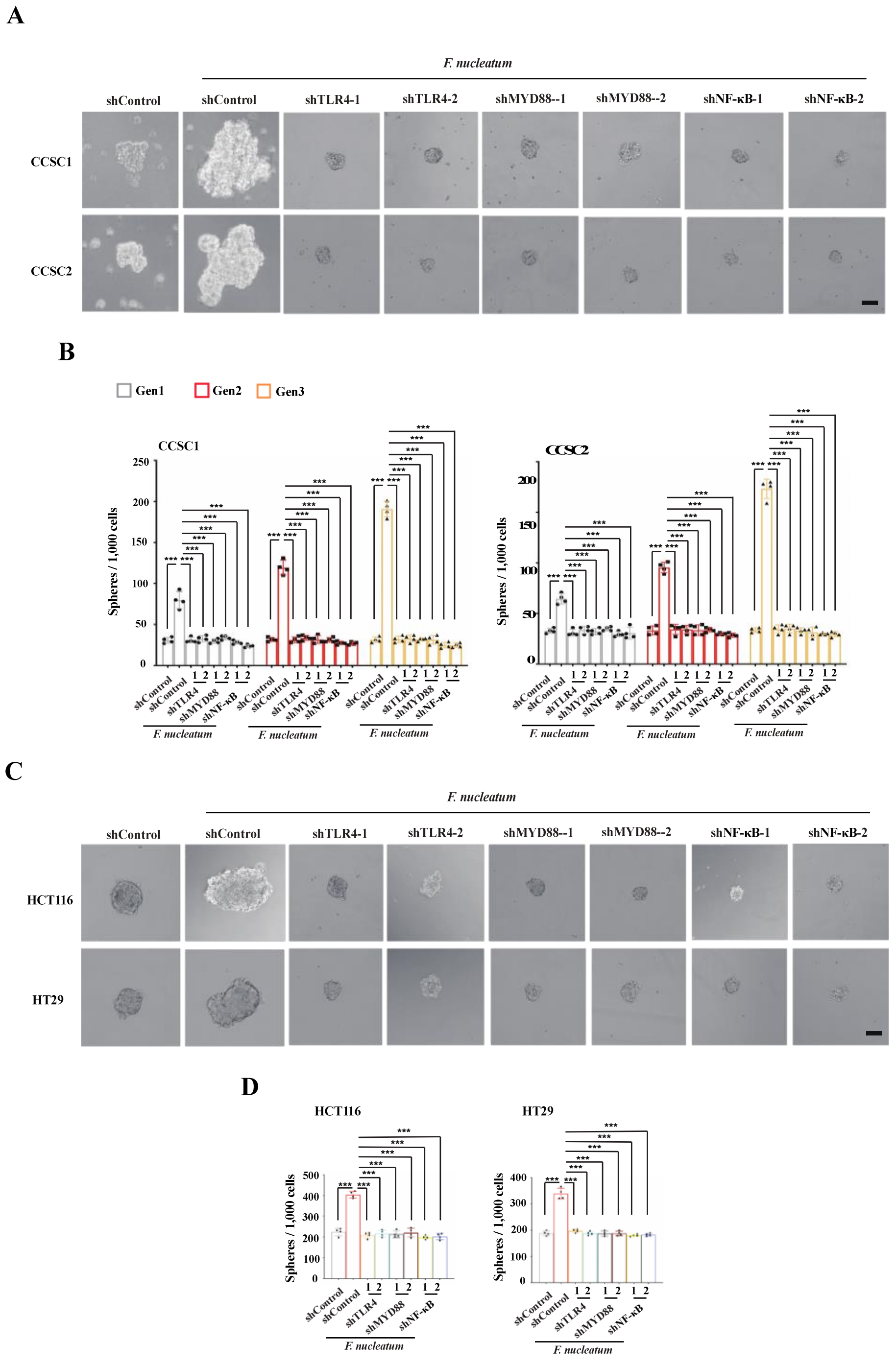

Figure S7. *F. nucleatum* upregulates sphere formation through NF-κB pathway.

(A and B) Representative images (A) and quantification (B) of sphere formation showing inhibition of NF-κB suppressed *F. nucleatum*-elevated sphere formation in

CCSCs. Error bars denote the s.d. (n = 4 per group). *P*-value was calculated using one-way ANOVA with post-hoc test. ***, $p < 0.001$. Scale bar, 100 μm.

(C and D) Representative images (C) and quantification (D) of sphere formation showing inhibition of NF-κB suppressed *F. nucleatum*-elevated sphere formation in HCT116 and HT29 cells. Error bars denote the s.d. (n = 4 per group). *P*-value was calculated using one-way ANOVA with post-hoc test. ***, $p < 0.001$. Scale bar, 100 μm.

Table S1. Sequences of shRNA and qPCR primers.

shRNA sequences

Name	Sense (5'-3')	Antisense (5'-3')
shAP2A-1	ccggacctccactggtgacagagaactcgagttctctgtcac cagtggaggttttttg	aattcaaaaaacctccactggtgacagagaactcgagttctct gtcaccagtggaggt
shAP2A-2	ccgggagcaaagaggcggaaattaactcgagttaatttccgc ctctttgctctttttg	aattcaaaaagagcaaagaggcggaaattaactcgagttaat ttccgcctctttgctc
shCPT1B-1	ccgggctgctaagaagcaccagaatctcgagattctggtgct tcttagcagctttttg	aattcaaaaagctgctaagaagcaccagaatctcgag attctggtgcttcttagcagc
shCPT1B-2	ccggccagatggagaggatgttcaactcgagttgaacatcct ctccatctggtttttg	aattcaaaaaccagatggagaggatgttcaactcgagttgaa catcctctccatctgg
shUBXD8-1	ccggcgcgcaaggttagcaacaaacctcgaggtttgttgcta accttgcgcgtttttg	aattcaaaaacgcgcaaggttagcaacaaacctcgaggtttg ttgctaaccttgcgcg
shUBXD8-2	ccggccacgacttcttattctccttctcgagaaggagaataag aagtcgtggtttttg	aattcaaaaaccacgacttcttattctccttctcgagaaggaga ataagaagtcgtgg
shFASN-1	ccgggctacgactacggccctcattctcgagaatgagggcc gtagtcgtagctttttg	aattcaaaaagctacgactacggccctcattctcgagaatgag ggccgtagtcgtagc
shFASN-2	ccggcgagagcacctttgatgacatctcgag atgtcatcaaaggtgctctcgtttttg	aattcaaaaacgagagcacctttgatgacatctcgag atgtcatcaaaggtgctctcg
shACSL3-1	ccgggctgtgtaacagttgtgaaatctcgagatttcacaactgt tacacagctttttg	aattcaaaaagctgtgtaacagttgtgaaatctcgagatttcac aactgttacacagc
shACSL3-2	ccgggccttcaagctgaaacgcaaactcgagtttgcgtttcag cttgaaggctttttg	aattcaaaaagccttcaagctgaaacgcaaactcgagtttgcg tttcagcttgaaggc
shRBPJk	ccgggcatgtagaaggaggtaatttctcgag aaattacctccttctacatgctttttg	aattcaaaaagcatgtagaaggaggtaatttctcgagaaatta cctccttctacatgc
shTLR4-1	ccggccgctggtgtatctttgaatactcgagtattcaaagatac accagcggtttttg	aattcaaaaaccgctggtgtatctttgaatactcgagtattcaaa gatacaccagcgg
shTLR4-2	ccggccaagtagtctagctttcttactcgag taagaaagctagactacttggtttttg	aattcaaaaaccaagtagtctagctttcttactcgagtaagaaa gctagactacttgg
shMYD88-1	ccggacagacaaactatcgactgaactcgag ttcagtcgatagtttgtctgt tttttg	aattcaaaaaacagacaaactatcgactgaactcgag ttcagtcgatagtttgtctgt
shMYD88-2	ccggcatcaagtacaaggcaatgaactcgag ttcattgccttgtacttgatgtttttg	aattcaaaaacatcaagtacaaggcaatgaactcgagttcatt gccttgtacttgatg
ShNF-kB-1	ccgggccttaatagtagggtaagttctcgag aacttaccctactattaaggctttttg	aattcaaaaagccttaatagtagggtaagttctcgagaacttac cctactattaaggc
ShNF-kB-2	ccggcggattgaggagaaacgtaaactcgagtttacgtttctc ctcaatccgtttttg	aattcaaaaacggattgaggagaaacgtaaactcgagtttac gtttctcctcaatccg

qPCR primer sequences

Name	sense (5'-3')	antisense (5'-3')
Lgr5	gagttacgtcttgcgggaaac	tgggtacgtgtcttagctgatta
Olfm4	actgtccgaattgacatcatgg	ttctgagcttccaccaaaactc
Sox9	agcgaacgcacatcaagac	ctgtaggcgatctgttgggg
Aldh1	gcacgccagacttacctgtc	cctcctcagttgcaggattaaag
Cdx2	gcagccaagtgaaaaccagg	tctcagagagccccagcg
CPT1B	gcgccccttgttggatgat	ccaccatgacttgagcaccag
FASN	cttgcaggagttctgggaca	ccgtccacgatggcttcata
Numb	aggccagtcgtccacatca	ggtacttaaccgggaagctacat
UBXD8	gacttacctggtgtcagaacgc	ctttctcctggtcagctctgag
AP2A	ctgcaagaagaacccagatga	tgggacgaagtagtaggtgtag
ACSL3	ctttctcacggatgccgcattg	ctgctgccatcagtgttggtttc
RBPJk	ggataggaaatagtgaccaagaaatg	agtgctttcgcttgtctgag
TLR4	ggatgaggactgggtaaggaatga	agcggctctggatgaagtgc
MYD88	gccgccggatggtggtggttgt	ttggtgcaggggttggtgtagtcg
NF-kB	atcccatctttgacaatcgtgc	ctggtcccgtgaaatacacctc
GAPDH	tgtagttgaggtcaatgaaggg	acatcgctcagacaccatg
Fusobacterium nucleatum	ggatttattgggcgtaaagc	ggcattcctacaaatatctacgaa
Universal Eubacteria 16S	ggtgaatacgttcccgg	tacggctaccttgttacgactt

Table S2. Antibodies.

Antibody	Species	Dilution	Source	Identifier
ALDH1	Rabbit	1:200 for IHC<br>1:20 for FACS	Abcam	ab52492
MDM2	Rabbit	1:1000 for WB<br>1:100 for ICC/IF	Abcam	ab259265
VCP	Rabbit	1:1000 for WB<br>1:100 for ICC/IF	Proteintech	10736-1-AP
AP2A	Rabbit	1:1000 for WB<br>1:100 for ICC/IF	Proteintech	11401-1-AP
Numb	Mouse	1:1000 for WB<br>1:100 for ICC/IF	Proteintech	60137-1-Ig
CPT1B	Rabbit	1:500	CUSABIO	CSB-PA821674LA01HU
FASN	Rabbit	1:1000	Proteintech	10624-2-AP
NICD	Rabbit	1:1000	CST	4147
Ubiquitin	Rabbit	1:1000	Proteintech	10201-2-AP
ADRP	Rabbit	1:1000	Abcam	ab108323
GAPDH	Mouse	1:1000	Abcam	ab8245
ACSL3	Rabbit	1:1000	Proteintech	20710-1-AP
TLR4	Rabbit	1:1000	Abcam	ab13867
MYD88	Rabbit	1:1000	Abcam	ab133739
P-P65	Mouse	1:1000	CST	3036
P65	Rabbit	1:1000	CST	4764
IκB-α	Rabbit	1:1000	CST	4812
β-actin	Rabbit	1:1000	CST	4967
HIF1α	Mouse	1:200	Proteintech	66730-1-Ig
PE-CD133	Mouse	1:200	Biolegend	Clone 7
APC-CD44	Mouse	1:200	Sungene Biotech	Clone HI44a
488-goat anti-mouse IgG	Goat	1:200	Life Technologies	A-11001
488-goat anti-rabbit IgG	Goat	1:200 for IHC<br>1:200 for FACS	Life Technologies	A-11008
HRP-goat anti-rabbit IgG	Goat	1:6000	EMAR	EM35111
HRP-goat anti-mouse IgG	Goat	1:6000	EMAR	EM35110
